# Cellular senescence and chronological age in various human tissues: A systematic review and meta‐analysis

**DOI:** 10.1111/acel.13083

**Published:** 2019-12-05

**Authors:** Camilla S. L. Tuttle, Mariette E. C. Waaijer, Monique S. Slee‐Valentijn, Theo Stijnen, Rudi Westendorp, Andrea B. Maier

**Affiliations:** ^1^ Department of Medicine and Aged Care Royal Melbourne Hospital University of Melbourne Melbourne Vic. Australia; ^2^ Department of Gerontology and Geriatrics Leiden University Medical Center Leiden The Netherlands; ^3^ Geriatric Rehabilitation Cordaan Amsterdam The Netherlands; ^4^ Department of Biomedical Data Sciences Leiden University Medical Center Leiden The Netherlands; ^5^ Department of Public Health and Centre for Healthy Ageing University of Copenhagen Copenhagen Denmark; ^6^ Department of Human Movement Sciences Faculty of Behavioural and Movement Sciences Amsterdam Movement Sciences Vrije Universiteit Amsterdam The Netherlands

**Keywords:** aging, cell cycle proteins, cellular senescence, human, immunosenescence

## Abstract

Senescent cells in tissues and organs are considered to be pivotal to not only the aging process but also the onset of chronic disease. Accumulating evidence from animal experiments indicates that the magnitude of senescence can vary within and between aged tissue samples from the same animal. However, whether this variation in senescence translates across to human tissue samples is unknown. To address this fundamental question, we have conducted a systematic review and meta‐analysis of all available literature investigating the magnitude of senescence and its association with chronological age in human tissue samples. While senescence is higher in aged tissue samples, the magnitude of senescence varies considerably depending upon tissue type, tissue section, and marker used to detect senescence. These findings echo animal experiments demonstrating that senescence levels may vary between organs within the same animal.

## Graphical abstract

Senescence load in human tissues is associated with chronological age, but heterogeneous between human tissues.

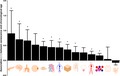

## 1 INTRODUCTION

Senescence, the process by which cells stop dividing and enter a state of permanent growth arrest, has been identified as a key hallmark of aging (López‐Otín, Blasco, Partridge, Serrano, & Kroemer, 2013). A higher number of senescent cells have not only been identified in tissue of older individuals (Wang et al., 2009), but also in individuals of higher biological age (Waaijer et al., 2015, 2012), in cancer patients undergoing chemotherapy (Demaria et al., 2017) and at sites of pathology of age‐related diseases (Childs, Durik, Baker, & van Deursen, 2015). Animal experiments have shown that senescent cell numbers can vary across tissue samples within one animal (Baker et al., 2016, 2011; Bussian et al., 2018; Krishnamurthy et al., 2004; Wang et al., 2009). This variation in cells expressing senescence markers may reflect different rates of tissue renewal and/or different triggers of senescence, within tissue types.

Seminal experiments that eliminate senescent cells in progeroid and aged mice demonstrate a markedly improved health span after senescence cells are cleared (Baker et al., 2016; Hickson et al., 2019; Jeon et al., 2017; Ogrodnik et al., 2019; Zhu et al., 2015). However, the clearance of senescent cells within and across different tissue samples from the same animal can be variable (Baker et al., 2016; Ogrodnik et al., 2019). Whether this variation in senescent cell clearance is a result of the treatment, age or disease is largely unknown. Currently, whether senescent cells accumulate at the same rate within and across aging tissue samples within humans is unknown. As such, to assess and collate the current evidence and understanding of senescence cells within aged human tissue samples, a systematic review and meta‐analysis of the current literature was conducted.

## 2 RESULTS

### 2.1 Selection of included studies

**Figure 1:**
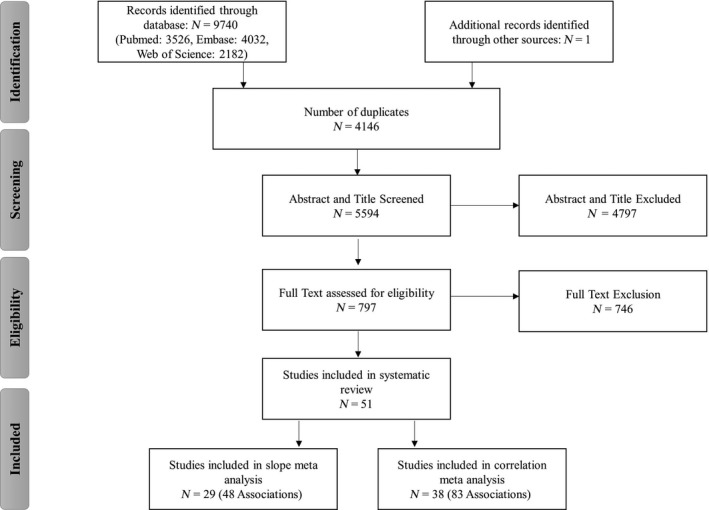
Overview of the search strategy

Figure 1 illustrates the literature search process. The search retrieved 9,740 articles. After exclusion of duplicates, 5,594 articles were screened for titles and abstracts of which 797 were screened for full text.

### 2.2 Characteristics of included studies

Overall, 51 articles were included in the systematic review. Table S1 provides a comprehensive overview of included articles. The total number of included participants was 2,072. Overall, most studies reported an age range rather than a mean/median age of the study population. As such, the age of participants included in this review ranged from neonatal to 120 years. Sex distributions of the study population were given in 33 of 51 articles (65%), in which 52% (*n* = 874/1,694) of the participants are female. Cell cycle regulators (*n* = 34/51 articles), particularly p16^INK4a^ (*n* = 27/51 articles), were the most used markers for detection of senescence in human tissue samples. The majority of articles (*n* = 36/51) used only one senescence marker to assess the relationship between senescence and chronological age. Overall, 14 different tissue types have been used to assess the relationship between senescence and age with skin (*n* = 12/51 articles), kidney (*n* = 11/51 articles), and blood (*n* = 6/51 articles) utilized most often. Eight articles that have utilized skin, brain, and kidney tissue have provided an in‐depth analysis of these samples based on tissue structure (e.g. dermis vs. epidermis) or cell type (e.g. astrocytes vs. oligodendrocytes).

### 2.3 Systematic review: qualitative description

Of the 51 articles assessed, 31 reported a significantly positive association between senescence and chronological age for at least one senescence marker while two describe a negative association between senescence and age. Eleven articles also reported a higher level of senescence with chronological age, but these findings were not statistically significant. In addition, while some articles (*n* = 7/51) described a positive association between senescence and chronological age, quantitative data to support this association have not been provided by the authors. Further information regarding the overall qualitative outcomes for each study can be found in Table S2.

### 2.4 Meta‐Analysis: Association between the magnitude of senescence and chronological age

**Figure 2:**
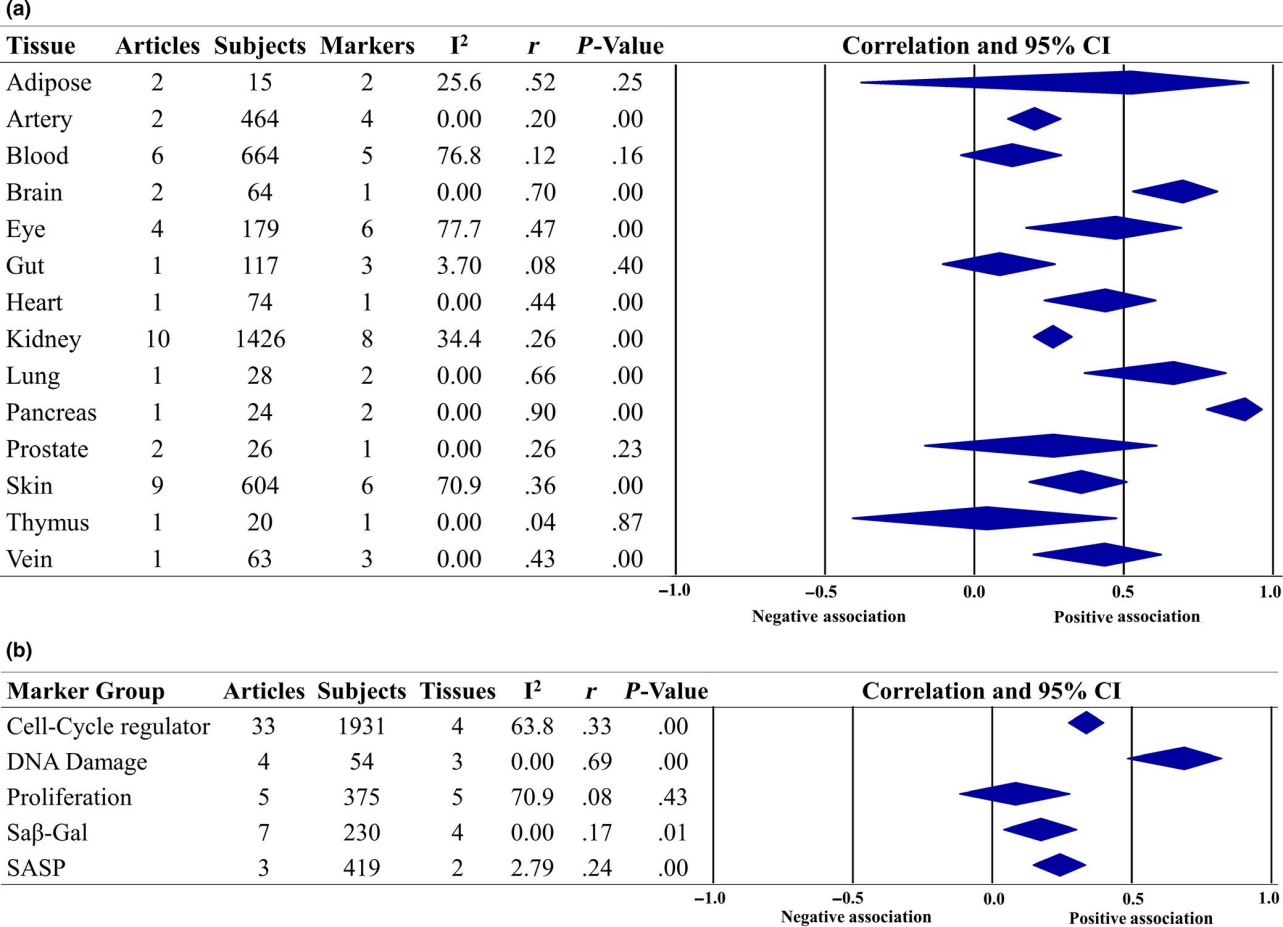
Overall forest plots of the meta‐analysis for senescence and chronological age subgrouped by tissue (a) and marker type (b)

Overall, 38 articles provided quantified data that could be used in the meta‐analysis, altogether describing 83 associations between senescence and chronological age. For the detailed meta‐analysis, including all articles and associations, refer to Figures S1 and S2. Figure 2 illustrates the overall correlation between senescence and age, subgrouped by tissue type (2A) and senescence marker type (2B). Overall, a positive correlation between senescence and age was observed (*r* = .28, *p* = .000, 95% prediction interval = −0.12 through 0.60, *τ* ^2^ = 0.042). Pancreas (*r* = .90, *p* < .001, one article, two markers, one tissue type), brain (*r* = .70, *p* < .001, two articles, one marker, four tissue types), and lung tissue (*r* = .66, *p* < .001, one article, two markers, one tissue type) showed the highest correlation between senescence and age. Of the different tissue types assessed, senescence in adipose, gut, prostate, and thymus tissues was not significantly correlated with age. Senescence markers identified as DNA damage markers had the highest correlation with age (*r* = .69, *p* < .001), followed by cell cycle regulators (*r* = .33 *p* < .001), whereas proliferation markers had the lowest correlation with age (*r* = .08, *p* = .43).

**Figure 3:**
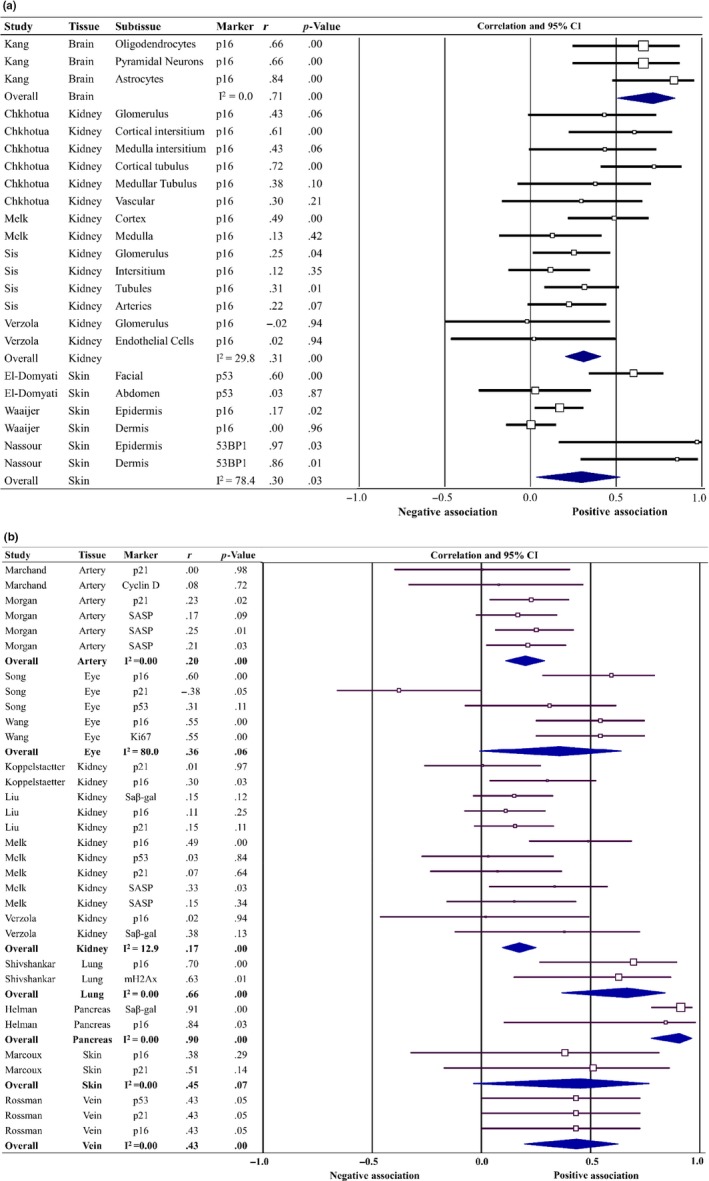
Forest plots of the subgroup meta‐analysis of senescence and chronological age for multiple associations extracted from the same article assessing senescence marker expression across tissue sections (a) and senescence marker expression within a tissue sample (b)

Figure 3a describes the association of senescence with age in multiple tissue sections within a specific tissue. Overall, a positive association (*r* = .31, *p* < .001), between senescence and age, was observed within the kidney tissue. However, while a positive association was observed between senescence and age within skin sections (dermis, epidermis, facial, and abdominal), significant heterogeneity was also observed (*r* = .30, *p* = .03, *τ* ^2^ = 0.067). The overall association between senescence and age within this analysis also demonstrated significant heterogeneity (*r* = .25, 95% prediction interval = −0.21 through 0.62, *τ* ^2^ = 0.049). Figure 3b describes the association of senescence with age where multiple senescent markers have been used in one tissue sample, the gold standard for detecting senescent cells within tissue samples (Gorgoulis et al., 2019). Overall, a positive association between senescence and age was observed irrespective of the marker used to determine senescence. However, significant heterogeneity was observed (*r* = .24, 95% prediction interval = −0.17 through 0.64, *τ* ^2^ = 0.040), and within one tissue sample, the expression of senescent markers varied (Song, Yang, Xie, Zang, & Yin, 2008).

### 2.5 Meta‐analysis: change in slope of senescence per 10 years of life

**Figure 4:**
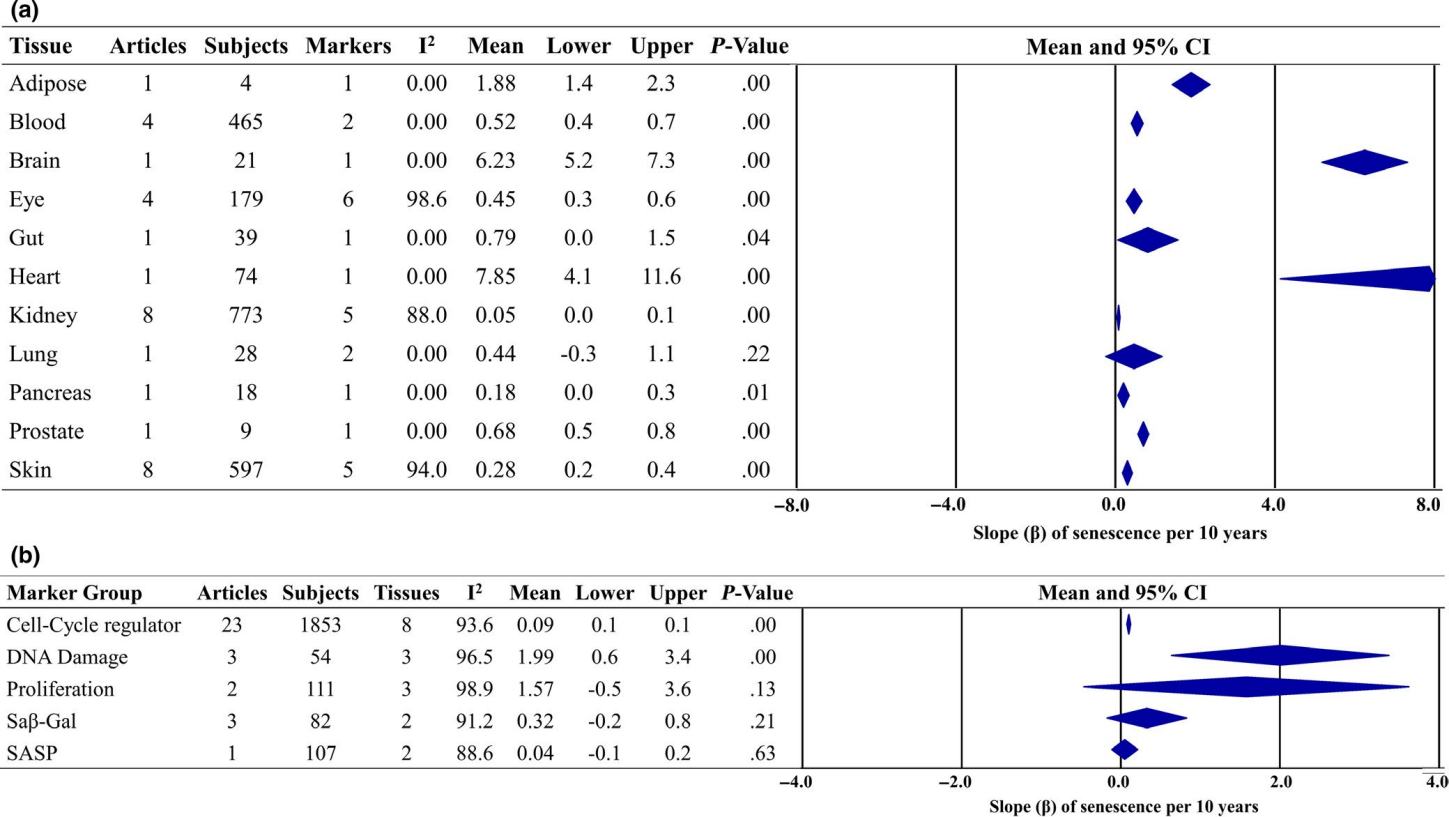
Overall forest plots of meta‐regression analyses for senescence load per 10 years of chronological age, expressed as the change in the overall slope (standardized units), subgrouped by tissue (a), and marker type (b)

To determine the magnitude of the change in senescence level with age, a meta‐analysis using mean change in *β* was performed. For the detailed meta‐analysis, including all articles and associations refer to Figures S3 and S4. Figure 4 illustrates the overall findings of the meta‐analysis subgrouped by tissue and marker type. Overall, there was a significantly higher level of senescence per 10 years of age across tissue samples; the combined overall effect size indicated a 0.16 ± 0.02 standardized unit (S.U) (*p* < .001, 95% prediction interval = 0.02–0.29, *τ* ^2^ = 0.0045) and higher magnitude of senescence per 10 years. The highest magnitude of senescence per 10 years of age was observed in brain (*β* = 6.23 ± 0.55 S.U) and adipose (*β* = 1.88 ± 0.24 S.U) tissue. Similarly, senescence markers identified as DNA damage markers were higher per 10 years of age (*β* = 1.99 ± 0.70 S.U), whereas the senescence‐associated secretory phenotype (SASP) had the lowest magnitude of senescence per 10 years of age (*β* = 0.04 ± 0.08 S.U). Significant heterogeneity (*I* ^2^ = 81.1%–98.6%) was observed for all tissue types and marker subgroups included in the meta‐analysis.

### 2.6 Publication bias

**Figure 5:**
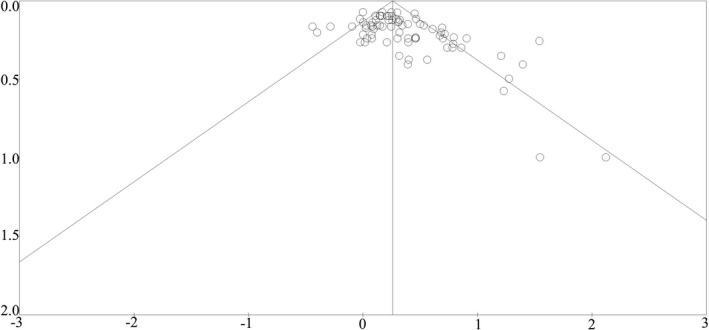
Begg's funnel plot for correlation meta‐analysis of published articles (*n* = 38)

Publication bias was assessed for all outcomes via visually inspecting the asymmetry of the funnel plot (Figure 5). The funnel plot including all data points demonstrated asymmetry. Smaller studies that do not show a positive association between senescent makers and age are underrepresented in this review. This, combined with Egger's regression intercept analysis, indicated publication bias toward the positive finding (*p* = .001).

## 3 DISCUSSION

This is the first study to quantify the association between the magnitude of senescence and chronological age across different human tissue types. A qualitative analysis of the literature identifies a largely positive association between cellular senescence and chronological age; however, the strength of the association differed based on the tissue type, subsection of tissue, and the senescence marker used within and between tissues.

The observed differences in the strength of the association between senescence and chronological age between tissue types may be explained by the natural cell turnover rate of tissue which is known to vary widely (Pelc, 1964). However, a tissue‐specific response to environmental exposures or different defense mechanisms against cytotoxic stress may also explain this variable senescence level within human tissue types. For example, Demaria and colleagues demonstrate higher numbers of senescence cells in patients who have received chemotherapy (Demaria et al., 2017). To date, there are little data available on senescence within different organ systems from the same individual (Dock et al., 2017; Skowronska‐Krawczyk et al., 2015). Articles included in this review that investigated the difference in senescence within tissues of the same organ showed that despite higher senescence within these tissues, the magnitude of senescence differed based on the tissue or cell type investigated (Chkhotua et al., 2003; Kang et al., 2015; Sis et al., 2007; Verzola et al., 2008). Dock and colleagues also investigated senescence within individuals using tissue samples from different organs (blood and gut) (Dock et al., 2017) and demonstrated that the magnitude of senescence was not only variable depending on the tissue assessed but also the marker used to define senescence. More recently, Chatsirisupachai and colleagues investigated the RNA‐Seq‐based gene expression of noncancerous tissues from humans and also reported higher and yet varying expression of senescence‐associated genes in aged tissue samples (Chatsirisupachai, Palmer, Ferreira, & de Magalhães, 2019). Thus, in alignment with the findings of this review, the current evidence would suggest that while cellular senescence is likely to increase with chronological age, the magnitude of senescence can vary from tissue to tissue. How this variation in senescence contributes to the onset of age‐related disease is yet to be determined.

This analysis did identify some tissue types (adipose, gut, prostate, and thymus) where senescence was not significantly associated with age. The lack of significance in these tissues could be caused by the limited number of studies investigating senescence and age within these tissues and the smaller sample sizes within these articles. Thus, despite positive correlations the relationship between senescence and age within these tissues requires confirmation through additional studies. On the other hand, adipose and thymus tissue are also postmitotic tissue. Senescence within postmitotic cells, such as neurons, adipose, and skeletal muscle, has been largely overlooked in human research, which is reflected in this current review. This is likely due to a lack of evidence as to whether postmitotic cells can become senescent (van Deursen, 2014). However, within the last decade a small number of studies have shown that postmitotic neurons in human and mouse brains accumulate high rates of DNA damage and exhibit additional senescent‐associated properties such as pro‐inflammatory cytokines and senescence‐associated beta galactosidase (SAβ‐gal) (Bhat et al., 2012; Jurk et al., 2012; Kang et al., 2015). More recently, the senescence phenotype has also been demonstrated within several other postmitotic tissues such as adipose (Minamino et al., 2009; Oubaha et al., 2016), osteoclasts and osteoblasts (Farr et al., 2016, 2017), cardiomyocytes (Anderson et al., 2019), and ganglion cells (Oubaha et al., 2016). Despite most of the postmitotic evidence existing within animal models, these findings demonstrate that postmitotic tissue is likely to become senescent and these tissue types should be considered when analyzing the magnitude of senescence within human populations.

In addition to heterogeneity of senescence between and within tissue samples, senescence varied widely depending on the marker used to detect senescence. Notably, correlation of senescence markers and age differed substantially for proliferation and DNA damage markers. This is thought to be caused by the various other cellular processes these markers are involved in, as proliferation and DNA damage are not specific to the senescent phenotype (Riffle, Pandey, Albert, & Hegde, 2017). Furthermore, production of SAβ‐gal does not necessarily indicate senescence either: Quiescent cells in culture are also known to express SAβ‐gal (Holt & Grainger, 2012). Thus, the higher expression of any senescent marker within tissue samples as evidence of senescence must be viewed with caution. These observations are supported here by the pronounced heterogeneity of senescence within the same tissue sample, such as skin and eye, using different senescence markers.

Recently, several molecules both previously identified (lamin B1) and newly identified (senescence‐associated distension of satellites (SADS) and senescence‐associated hematochromatin foci (SAHF)) have also been recognized as markers of senescence in vivo. Animal models have shown lamin B1, a protein forming part of the CeD‐3/ICE family that is essential to initiating processes of apoptosis within a cell, to be downregulated within senescent cells (Freund, Laberge, Demaria, & Campisi, 2012). On the other hand, SADS, that is centromere decondensation that occurs within the early stages of senescence, can be measured in several ways including the upregulation of SIRT6 which is key to satellite distension (Freund et al., 2012). The detection of SADS may be a more robust method for detecting senescent cells than the current senescent markers used. The growing family of markers of senescence has been recently reviewed (Gorgoulis et al., 2019; Hernandez‐Segura, Nehme, & Demaria, 2018). It will be important that these markers be trialed within human tissues to determine how robust they are in detecting senescent cells within human populations. The current available senescence markers all have their caveats and restrictions, and currently, there is not a universal marker to detect senescent cells (Sharpless & Sherr, 2015). Until a universally accepted marker is established, a cluster of senescent markers, focusing on a specific senescent pathway (reviewed in (Childs et al., 2015)), should be used to validate senescence within human tissue samples.

Despite the significant heterogeneity, overall the finding of this review indicates a positive relationship between senescence and chronological age. However, it is important to note that all studies presented within this review are cross‐sectional in nature. Therefore, the observed results cannot be interpreted as an increase in senescence within human tissue of an individual as they age. However, there is strong evidence from in vitro and animal research indicating not only a causal relationship between senescence and age (Baker et al., 2016; Wang et al., 2009) but that senescence increases with older age as a result of a positive feedback loop of the senescent phenotype (Kandhaya‐Pillai et al., 2017; Passos et al., 2010). Alternatively, a higher number of senescent cells may not be due to an increased production of senescence, but rather a reduced rate of clearance of senescent cells. Evidence from animal studies has identified a role for the innate immune system (macrophages, NK cells) to remove senescent cells in premalignant lesions (Kang et al., 2011; Xue et al., 2007). Thus, an impaired maintenance of the senescent cell equilibrium (generation and clearance) may also explain the positive association between senescence and chronological age observed here. While biological processes may be one explanation for our findings, publication bias must also be considered. Studies with small sample sizes, that is most articles reported here, and negative results are more prone to publication bias (Song, Eastwood, Gilbody, Duley, & Sutton, 2000; Sterne, Gavaghan, & Egger, 2000). The asymmetry of the generated funnel plot analysis for the included articles indicates a positive publication bias. As such, the overall strength of the association between senescence load and chronological age observed within the meta‐analyses should be interpreted judiciously.

The research reported here is not without limitations. The design of the study aimed to include all examined markers of senescence (except telomere length, reviewed in von Zglinicki and Martin‐Ruiz (2005)) within the isolated articles irrespective of whether they were measured within the same population. This has led to some articles contributing multiple associations within the meta‐analysis. This is likely to have a positive bias on the power and significance of the overall meta‐analysis. On the other hand, some articles could not be included in this analysis due to insufficient data reported. In addition, age range was not an exclusion criterion for this review; hence, the included populations ranged from neonatal to centenarian. The role of senescent cells during the development, reproductive, and postreproductive phase of life may differ significantly, and a linear association between senescence and age may not be an adequate interpretation of this relationship. Furthermore, in addition to using different markers to detect senescence levels within aged tissue, various molecular techniques (e.g. IHC, qPCR, and flow cytometry) to detect senescence have been utilized. The mRNA expression of a marker of senescence may differ from the protein expression of the same senescence marker within and between tissue samples. How these findings are reported (percentage of senescence cells in a tissue section vs. the magnitude of staining) also impacts the reported magnitude of senescence between studies. Thus, the technical approach to assess senescence as well as the theoretical approach is likely to have contributed to the overall heterogeneity reported in this analysis.

This study has also identified important variables that have been overlooked in this field of research. In this review, we have included all articles that have identified senescence irrespective of whether the senescence phenotype has been verified with additional senescence markers. To exclude papers where only one marker of senescence was used to detect senescence would not provide an overview of the current state of the literature. However, the use of one senescence marker to detect senescence does not equate to a senescent cell, as these markers are not specific to senescence pathways (Sharpless & Sherr, 2015). Furthermore, the upregulation of one senescence marker may not act as a surrogate for another marker. For example, the p53‐p21 CDK inhibitors are strongly associated with each other in downstream senescence pathways. However; Song and colleagues demonstrate higher p53 but lower p21 in aged corneal cells. The role of p53 in senescence and aging has been previously reviewed and does not always involve the p21 pathway (Rufini, Tucci, Celardo, & Melino, 2013). This review and meta‐analysis highlights that thus far within this field, senescence has been established not by a senescence phenotype but rather the increase or decrease of one senescence marker. Moving forward, senescence cells within humans should be confirmed via the use of several senescence markers, indicating a senescence phenotype (Gorgoulis et al., 2019). Not only is it essential that more than one known senescent marker be used to identify senescence within a tissue type—it is also important that these markers be assessed within the same experimental specimen, that is, through experimental techniques such as multiple immunofluorescent staining, as this detects the colocalization of these markers within a cell. In addition, it is important that researchers report precisely on the strength and variances of tested associations between senescence and age using an appropriate statistical test. Several articles lacked information for the subject demographics and characteristics from whom the tissues were sampled, which made interpretation of findings difficult, given the reported differences between senescence markers and sex (Ostan et al., 2016).

The findings of this systematic review and meta‐analysis support the hypothesis that senescence is higher within and across tissue samples as human's age. However, the magnitude of senescence may vary between tissues, within tissues, and depending on the marker used to detect senescence. These findings echo the findings of animal experiments that demonstrate variation in senescence within and between tissue samples from the same animal.

## 4 METHODS

### 4.1 Selection of articles

A systematic search of the literature was performed on January 31, 2018, in PubMed, EMBASE, and Web of Science using the terms “senescence,” “tissue/biopsy/histology,” different “organ/tissue types,” and different “markers for cellular senescence” (see Appendix S1). Titles and abstracts were screened by reviewers (MW, MS, and CT) to exclude irrelevant articles. Exclusion criteria were defined as follows: (a) animal studies; (b) in vitro studies; (c) reviews; (d) conference abstracts; (e) method/theory papers (without primary data); (f) editorials; (g) tissue samples from diseased organs (note: Healthy tissue outside area of pathology was included); and (h) articles written in a language other than English. After title and abstract screening, full text of the remaining articles was screened by reviewers (MW, MS, and CT) using the aforementioned exclusion criteria and the following additional criteria: (i) methodology to detect senescent cells not reported; (j) no association between the presence of senescent cells and age described; (k) the presence of senescent cells in cancer tissue; and (l) telomere length or markers used to assess immunosenescence was the only reported marker of senescence. Telomere length was excluded as a marker of senescence because sensitivity and specificity of telomere length as a marker of cellular senescence have been reported to be low (Sharpless & Sherr, 2015), and the causal relationship of telomere attrition to cellular senescence in vivo has not been established (reviewed in Cristofalo, Lorenzini, Allen, Torres, & Tresini, 2004; Sharpless & Sherr, 2015). Markers of immunosenescence were excluded because although immunosenescence refers to the decline of the immune system with age; the shift from naïve to memory states cell types does not necessarily equate to senescence (Larbi & Fulop, 2014). Whether these cells have become senescent can only be established with the use of additional senescence markers. Any disagreement between reviewers was arbitrated by a fourth reviewer (AM).

### 4.2 Extraction of data

Data were extracted for this review by two reviewers (MW and CT). Using a standardized data extraction form, the following information was extracted: type and origin of tissue; the number, chronological age, and gender of the subjects; and the marker(s) used to determine senescence, technique used, association between the senescence markers and age (including raw data points for individual subjects), and statistical tests used for analysis. In addition, senescence markers were further categorized into “type of markers” as determined by the markers' role within the cell cycle and senescence. For analysis purposes, the reported direction of markers, known to be negatively associated with senescence, for example proliferation markers, was reversed. If an article reported on the senescence load of more than one tissue type within a population, this information was extracted as separate associations in order to establish if the association between senescence load and age was consistent across and within tissue samples. Similarly, to determine whether the association between senescence marker and age was also consistent, if an article reported more than one senescence marker, these data were extracted as separate associations. As such, the number of associations used in this meta‐analysis is higher than the number of articles.

### 4.3 Meta‐analysis

#### 4.3.1 Meta‐analysis: association between senescence and age

The correlation meta‐analysis was performed using Comprehensive Meta‐Analysis (CMA) software (Biostat). To determine the overall association between cellular senescence and age, data extracted from articles were entered in two formats, depending on data availability. Where the article provided a correlation coefficient (*r*) for senescence load and age, *r*, sample size, and direction of the association were used, where authors did not provide *r* but provided sample size, *p* value, and direction of the association, these data were entered in to the CMA program, and *r* was calculated. *Z*‐scores were calculated from the correlation coefficients using Fisher's method and pooled under a random‐effects model. The pooled *Z*‐scores were then back‐transformed to the pooled correlation coefficients. Heterogeneity of the results was described using both the *I*‐squared and Tau‐squared coefficients. To determine the overall association between senescence load and age, the data were analyzed via tissue type and senescence marker used to determine senescence load. To describe the heterogeneity in the reported correlations between studies, the 95% prediction interval (95% PI) was calculated. This interval covers approximately 95% of the true correlations. In addition to the overall analysis, the meta‐analysis was subgrouped by tissue type and senescence marker. Due to the use of multiple tissue sections and multiple senescent markers, a study could be used twice or more within the same meta‐analysis. The overall results were not corrected for the dependence caused by the repeated population use. However, a further subgroup analysis was performed for articles that detected senescence load in multiple tissue sections within a tissue type and senescence load using multiple senescence markers within one tissue. A funnel plot and Egger's regression intercept were used to determine any indication of publication bias.

#### 4.3.2 Meta‐analysis: change in slope of senescence per 10 years of life

To determine the magnitude of the association of senescence markers per 10 years of age, the following extraction methodology was used to calculate the slope (i.e. the degree of change in the dependent variable (senescence markers) for every unit of change in the independent variable (10 years)). For continuous data: If the slope of senescence marker (*β*) was given per year, this was recalculated for a change per 10 years of age, plus the standard error. If a (Pearson) correlation coefficient (*r*) was given and the spread of data points (standard deviation (*SD*)) on the *x*‐axis (SDx) and *y*‐axis (SDy) could be derived from a plot (by calculating the *SD* from data points in the plot, assuming normal distribution). The formula *r* = *β* (SDx/SDy) was used to calculate the slope of senescence marker per 10 years. In case of comparisons of age groups, mean senescence marker per age‐group with standard error was recorded. Variance‐weighted least‐squares regression in Stata 12 (Stata Statistical Software: Release 12: StataCorp. LP) was used to calculate the slope of senescence marker per 10 years of age. To standardize the different senescence markers and/or detection methods used, the slope of the senescence marker was standardized based on the standard deviation of the senescence marker at the mean, median age, or middle age‐group (as provided in the article or as calculated from data plots). This resulted in slopes of senescence markers that could be pooled regardless of used markers or detection methods. Through this method, we add information on the magnitude of association between senescence load and age, rather than only the correlation coefficient. Studies that reported fold changes of senescence markers but gave no other information could not be included in this meta‐regression. The final data for the meta‐regression were imported into the Comprehensive Meta‐Analysis software (Biostat). To determine the overall change in senescence load per 10 years, the data were analyzed via tissue type and senescence marker used to determine senescence load. To describe the heterogeneity between studies in the reported slope change, the 95% prediction interval (95% PI) was calculated. This interval covers approximately 95% of the true slope change.

## CONFLICT OF INTEREST

The authors declare that they have no conflicts of interest.

## AUTHORS' CONTRIBUTION

MW, MS, TS, RW, and AM designed this review. MW, MS, and CT conducted the search and reviewed the articles returned by the search for eligibility. MW, MS, and CT completed data extraction, and MW and CT performed the meta‐analysis. MW and CT prepared the draft of this manuscript. AM oversaw the project and provided feedback on the search. All authors contributed to the writing and editing of the manuscript. MW and CT contributed equally to this work.

## Data Availability

We believe this paper is exempt under the following rule – revisions of papers originally submitted prior to the policy's adoption.
