# Supplementary material for: Cellular senescence and chronological age in various human tissues: A systematic review and meta‐analysis
Source: Aging Cell. 2019 Dec 5;19(2):e13083. doi: 10.1111/acel.13083 (PMC6996941; doi:10.1111/acel.13083)

**Appendix S1**

**Supplementary Information**

**Search Strategy**

***PubMed***

("senescent"[all fields] OR "senescence"[all fields] ) AND ("Tissues"[Mesh] OR "tissues"[all fields] OR "tissue"[all fields] OR "cell"[tiab] OR "cells"[tiab] OR "Biopsy"[Mesh] OR "Biopsy"[all fields] OR "Autopsy"[Mesh] OR "Autopsy"[all fields] OR "Histology"[Mesh] OR "Histology"[all fields] OR "histologic"[all fields]) AND ("Brain"[Mesh] OR "brain"[tiab] OR "Eye"[Mesh] OR "eye"[tiab] OR "eyes"[tiab] OR "cornea"[tiab] OR "lens"[tiab] OR "retina"[tiab] OR "retinal"[tiab] OR "Endocrine Glands"[Mesh] OR "thyroid"[tiab] OR "parathyroid"[tiab] OR "Cardiovascular System"[Mesh] OR "heart"[tiab] OR "myocard"[tiab] OR "myocardial"[tiab] OR "cardiovascular"[tiab] OR "artery"[tiab] OR "arteries"[tiab] OR "arterial"[tiab] OR "vein"[tiab] OR "venous"[tiab] OR "endothelial"[tiab] OR "Skin"[Mesh] OR "skin"[tiab] OR "dermis"[tiab] OR "dermal"[tiab] OR "epidermis"[tiab] OR "epidermal" OR "adipose"[tiab] OR "Lung"[Mesh] OR "lung"[tiab] OR "Lymphoid Tissue"[Mesh] OR "thymus"[tiab] OR "Breast"[Mesh] OR "breast"[tiab] OR "mammae"[tiab] OR "Digestive System"[Mesh] OR "intestinal"[tiab] OR "esophagus"[tiab] OR "esophagal"[tiab] OR "gastric"[tiab] OR "liver"[tiab] OR "pancreas"[tiab] OR "pancreatic"[tiab] OR "Urogenital System"[Mesh] OR "bladder"[tiab] OR "kidney"[tiab] OR "renal"[tiab] OR "adrenal"[tiab] OR "spleen"[tiab] OR "prostate"[tiab] OR "prostatic"[tiab] OR "uterus"[tiab] OR "uterine"[tiab] OR "ovaries"[tiab] OR "ovarian"[tiab] OR "testes"[tiab] OR "testicular"[tiab] OR "Muscles"[Mesh] OR "muscle"[tiab] OR "muscular"[tiab]) AND ("p16INK4a"[all fields] OR "Cyclin-Dependent Kinase Inhibitor p16"[Mesh] OR "Cyclin-Dependent Kinase Inhibitor p21"[Mesh] OR "p21"[all fields] OR "p21cip1"[all fields] OR "p21:MIB-1"[all fields] OR "p21WAF1/Cip"[all fields] OR "Tumor Suppressor Protein p53"[Mesh] OR "p53"[all fields] OR "GLB1 protein, human" [Supplementary Concept] OR "beta-galactosidase"[all fields] OR "SA-βgal"[all fields] OR "SA-betagal"[all fields] OR "H2AFX protein, human" [Supplementary Concept] OR "γH2AX"[all fields] OR "phospho-Histone H2A.X"[all fields] OR "γH2A.X"[all fields] OR "senescence-associated secretory phenotype"[all fields] OR "SASP"[all fields] OR "Ki-67 Antigen"[Mesh] OR "proliferation arrest"[all fields] OR "Ki-67"[all fields] OR "phospho-checkpoint 2 kinase"[all fields] OR "phospho-Chk2"[all fields] OR "Chromatin Assembly and Disassembly"[Mesh] OR "chromatin remodelling"[all fields] OR " senescence associated heterochromatic foci"[all fields] OR "senescence associated heterochromatic foci"[all fields] OR "SAHF"[all fields] OR "DNA damage response"[all fields] OR "DDR"[tiab] OR "DNA damage foci"[all fields] OR "DNA damage focus"[all fields] OR "Telomere"[Mesh] OR "Telomere Shortening"[Mesh] OR "Telomere Homeostasis"[Mesh] OR "telomere"[all fields] OR "telomeres"[all fields] )

***EMBASE***

("senescent".ti,ab. OR "senescence".ti,ab. ) AND (exp *tissues/ OR "tissues".ti,ab. OR "tissue".ti,ab. OR "cell".ti,ab. OR "cells".ti,ab. OR exp *biopsy/ OR "Biopsy".ti,ab. OR *autopsy/ OR "Autopsy".ti,ab. OR exp *histology/ OR "Histology".ti,ab. OR "histologic".ti,ab.) AND ( exp *Brain/ OR "brain".ti,ab. OR exp *Eye/ OR "eye".ti,ab. OR "eyes".ti,ab. OR "cornea".ti,ab. OR "lens".ti,ab. OR "retina".ti,ab. OR "retinal".ti,ab. OR exp *endocrine system/ OR "thyroid".ti,ab. OR "parathyroid".ti,ab. OR exp *Cardiovascular System/ OR "heart".ti,ab. OR "myocard".ti,ab. OR "myocardial".ti,ab. OR "cardiovascular".ti,ab. OR "artery".ti,ab. OR "arteries".ti,ab. OR "arterial".ti,ab. OR "vein".ti,ab. OR "venous".ti,ab. OR "endothelial".ti,ab. OR exp *Skin/ OR "skin".ti,ab. OR "dermis".ti,ab. OR "dermal".ti,ab. OR "epidermis".ti,ab. OR "epidermal" OR "adipose".ti,ab. OR exp *Lung/ OR "lung".ti,ab. OR exp *Lymphoid Tissue/ OR "thymus".ti,ab. OR exp *Breast/ OR "breast".ti,ab. OR "mammae".ti,ab. OR exp *Digestive System/ OR "intestinal".ti,ab. OR "esophagus".ti,ab. OR "esophagal".ti,ab. OR "gastric".ti,ab. OR "liver".ti,ab. OR "pancreas".ti,ab. OR "pancreatic".ti,ab. OR exp *Urogenital System/ OR "bladder".ti,ab. OR "kidney".ti,ab. OR "renal".ti,ab. OR "adrenal".ti,ab. OR "spleen".ti,ab. OR "prostate".ti,ab. OR "prostatic".ti,ab. OR "uterus".ti,ab. OR "uterine".ti,ab. OR "ovaries".ti,ab. OR "ovarian".ti,ab. OR "testes".ti,ab. OR "testicular".ti,ab. OR exp *Muscle/ OR "muscle".ti,ab. OR "muscular".ti,ab.) AND ("p16INK4a".ti,ab. OR *cyclin dependent kinase inhibitor 2A/ OR "Cyclin-Dependent Kinase Inhibitor p16".ti,ab. OR *cyclin dependent kinase inhibitor 1A/ OR "p21".ti,ab. OR "p21cip1".ti,ab. OR "p21:MIB-1".ti,ab. OR "p21WAF1/Cip".ti,ab. OR Protein p53/ OR "p53".ti,ab. OR *beta galactosidase/ OR "beta-galactosidase".ti,ab. OR "SA-βgal".ti,ab. OR "SA-betagal".ti,ab. OR "γH2AX".ti,ab. OR "phospho-Histone H2A.X".ti,ab. OR "γH2A.X".ti,ab. OR "senescence-associated secretory phenotype".ti,ab. OR "SASP".ti,ab. OR *Ki-67 Antigen/ OR "proliferation arrest".ti,ab. OR "Ki-67".ti,ab. OR "phospho-checkpoint 2 kinase".ti,ab. OR "phospho-Chk2".ti,ab. OR exp *"Chromatin Assembly and Disassembly"/ OR "chromatin remodelling".ti,ab. OR "senescence associated heterochromatic foci".ti,ab. OR "senescence associated heterochromatic foci".ti,ab. OR "SAHF".ti,ab. OR "DNA damage response".ti,ab. OR "DDR".ti,ab OR "DNA damage foci".ti,ab. OR "DNA damage focus".ti,ab. OR *Telomere/ OR *Telomere Shortening/ OR *Telomere Homeostasis/ OR "telomere".ti,ab. OR "telomeres".ti,ab. )

2960 op 12 mei 2014

***Web of Science***

TS= (senescent OR senescence) AND TS=(tissues OR tissue OR cell OR cells OR Biopsy OR Autopsy OR Histology OR histologic) AND TI=(brain OR eye OR eyes OR cornea OR lens OR retina OR retinal OR thyroid OR parathyroid OR heart OR myocard OR myocardial OR cardiovascular OR artery OR arteries OR arterial OR vein OR venous OR endothelial OR skin OR dermis OR dermal OR epidermis OR epidermal OR adipose OR lung OR Lymphoid OR thymus OR breast OR mammae OR intestinal OR esophagus OR esophagal OR gastric OR liver OR pancreas OR pancreatic OR bladder OR kidney OR renal OR adrenal OR spleen OR prostate OR prostatic OR uterus OR uterine OR ovaries OR ovarian OR testes OR testicular OR muscle OR muscular) AND TS= (p16INK4a OR cyclin dependent kinase inhibitor 2A OR Cyclin-Dependent Kinase Inhibitor p16 OR cyclin dependent kinase inhibitor 1A OR p21 OR p21cip1 OR p21:MIB-1 OR p21WAF1/Cip OR p53 OR beta-galactosidase OR SA-βgal OR SA-betagal OR γH2AX OR phospho-Histone H2A.X OR γH2A.X OR senescence-associated secretory phenotype OR SASP OR proliferation arrest OR Ki-67 OR phospho-checkpoint 2 kinase OR phospho-Chk2 OR chromatin remodelling OR senescence associated heterochromatic OR SAHF OR DNA damage response OR DDR OR DNA damage foci OR DNA damage focus OR telomere OR telomeres)

**Supplementary Tables**

| Supplementary Table 1. Population demographics of included articles ordered by tissue type. | | | | | | | | |
| --- | --- | --- | --- | --- | --- | --- | --- | --- |
| First author, Year | **Population** | | | | | **Origin** | **Senescence marker** | **Technique** |
|  | **G** | **N** | | **%M** | **Age (years)** |  |  |  |
| Adipose |  | | |  |  |  |  |  |
| Justice, 2017 | **-** | 11 | | 0 | µ 72.9, SD 3.4 | Deep to fascia, thigh | p16 | IHC |
| Xu, 2015 | Y | 4 | | 100 | µ 31, SD 5 | Femoral s.c. fat | Ki67, γH2AX | IHC |
|  | O | 4 | | 100 | µ 71, SD 2 |  |  |  |
| Artery |  | | |  |  |  |  |  |
| Marchand, 2011 | M | 11 | | 100 | µ 54.8, SE 5.5 | Waste product; internal mammary arteries | p21, cyclin D | WB |
|  | O | 13 | | 100 | µ 77.1, SE 2.2 |  |  |  |
| Morgan, 2013 | Y | 36 | | 52 | µ 31.2, SE 1.3 | Arterial biopsy; sentinel lymph node | p21, SASP (IL-6, IL-8, MCP-1) | PCR |
|  | O | 25 | | 63 | µ 70.9, SE 1.4 |  |  |  |
| Rossman, 2017 | Y | 18 | | 72 | µ 23, SE 1 | Arterial endothelial cells; antecubital veins | p16, p21, p53, SAβ-gal | IHC |
|  | OS | 30 | | 70 | µ 62, SE 1 |  |  |  |
|  | OT | 27 | | 74 | µ 59, SE 1 |  |  |  |
| Blood |  |  | |  |  |  |  |  |
| Schonland, 2003 | **-** | 74 | | nr | Range 24.4 - 90.1 | Healthy donors | Ki67 | FC |
| Liu, 2009 | E | 80 | | 45 | Range 18 - 80 | Healthy donors | p16 | qPCR |
|  | V | 90 | | 29 | Range 18 - 80 |  |  |  |
| Werner, 2009 | Y | | 26 | 57 | µ 21.8, SD 2.8 | Healthy donors | p53 | WB |
|  | O | | 21 | 66 | µ 50.9, SD 7.6 |  |  |  |
| Sanoff a, 2014 | **-** | | 176 | 0 | x̄ 67, R 50-93 | Breast cancer survivors | p16 | PCR |
| Sanoff b, 2014 | **-** | | 33 | 0 | Range 32 - 69 | Adjuvant breast cancer patients | p16 | PCR |
| Pustavoitau, 2015 | **-** | 47 | | 75 | x̄ 63.5, R 56 - 81 | Coronary artery bypass patients | p16 | qPCR |
|  |  |  | |  |  |  |  |  |
|  |  |  | |  |  |  |  |  |
| Brain |  |  | |  |  |  |  |  |
| Bhat, 2012 | **-** | 21 | | 40 | Range: Fetal - 90 | Frontal cortex and cerebellum; PM | p16 | IF |
| Kang, 2016 | Y | 8 | | nr | Young | Prefrontal cortical grey matter; healthy or neurodegenerative disease (normal neuropathology) PM | p16 | IF |
|  | O | 8 | | nr | Old |  |  |  |
| Eye |  |  | |  |  |  |  |  |
| Song, 2008 | **-** | 27 | | nr | Range 19 - 61 | Corneal rims; PM | p16, p21, p53 | PCR |
| Abdouh, 2012 | **-** | 9 | | nr | Range <30 - 75 | Retina; PM | BMI1, p16 | WB, IF, IHC |
| Zang, 2012 | **-** | 33 | | nr | x̄ 37, IQR 24 - 51 | Cornea; PM | p16 | PCR |
| Cao, 2013 | Y | 11 | | 36 | x̄ 48.0,  IQR 45.5 - 55.0 | Retinal pigment epithelial; PM | SASP (IL1-RA) | IHC |
|  | O | 12 | | 83 | x̄ 72.5,  IQR 72.0 - 74.0 |  |  |  |
| Gastrointestinal |  |  | |  |  |  |  |  |
| Going, 2002 | **-** | 46 | | nr | x̄ 69.5,  Range 29 - 90 | Oesophagus, stomach, duodenum | SAβ-gal | IHC |
| Helman, 2016 | **-** | 18 | | nr | Range 5 months – 60 years | Live islet cells; pancreas | p16, SAβ-gal | qPCR, IHC, FC |
| Dock, 2017 | **-** | 39 | | 92 | µ 40.8 | Rectosigmoid mucosal cells and blood | Ki67/CD4/CD8/CD45RA/CD28 | FC |
| Heart |  |  | |  |  |  |  |  |
| Kajstura, 2010 | **-** | 74 | | 57 | Range 19 - 104 | Left ventricle; PM CVD not cause of death | p16 | IF |
| Kidney |  |  | |  |  |  |  |  |
| Chkhotua, 2003 | **-** | 20 | | 70 | µ 54.4, SD 18 | Cortex and medulla; nephrectomies and renal transplants | p16 | IHC |
| Melk, 2004 | **-** | 42 | | 55 | x̄ 51.6, Range 8 weeks - 88 years | Cortex and medulla; nephrectomies, renal transplants & PM | p16, p53, p21, SASP (MMP-1, TGFβ1) | PCR |
| Sis, 2007 | Y | 19 | | 42 | µ 36.4, SD 9.6 | Glomeruli, interstitium, tubules, arteries,include diseased kidneys | p16 | IHC |
|  | M | 12 | | 17 | µ 52.8, SD 20.5 |  |  |  |
|  | D | 35 | | 66 | µ 47.9, SD 19.5 |  |  |  |
| Koppelstaetter, 2008 | **-** | 54 | | 69 | µ 46.3, SD 16.0 | Renal transplant | p21 | PCR |
| Verzola, 2008 | **-** | 17 | | 82 | µ 61, SE 2 | Glomeruli and tubules; nephrectomies | p16, SAβ-gal | IHC |
| McGlynn, 2009 | **-** | 73 | | 51 | µ 46.9, SD 16.3 | Cortex; renal transplants and PM | p16 | PCR |
| Liu, 2012 | **-** | 108 | | 54 | µ 35, SD 11.9 | Renal resection | p16, p21, SAβ-gal | IHC |
| Gingell-Littlejohn, 2013 | **-** | 33 | | 45 | µ 48, SD 15.7 | Renal; PM | p16 | PCR |
| Berkenkamp, 2014 | **-** | 58 | | nr | Range 17.8 - 93.3 | Healthy, nephrectomies and renal transplants | Cyclin D | IHC |
| Gunther, 2017 | **-** | 60 | | 60 | µ 48.4, SD 17.2 | Renal transplants | CDKN2A, | qPCR |
| Lung |  |  | |  |  |  |  |  |
| Shivshankar, 2011 | **-** | 14 | | nr | Range 43 - 82 | No pathology present | p16, mH2A | WB |
| Disayabutr, 2016 | **-** | 10 | | 50 | µ 40, SD 19 | Type II AECs; explanted human lungs & controls | SAβ-gal | FC |
| Prostate |  |  | |  |  |  |  |  |
| Choi, 2000 | **-** | 43 | | 100 | x̄ 62.0,  IQR 57.5 - 66.0 | Transitional zones prostatectomy and transrectal ultrasound | SAβ-gal | IHC |
| Castro, 2003 | **-** | nr | | 100 | Range 40 - >70 | Benign tissue hyperplastic transition zone; prostatectomy | SAβ-gal | IHC |
|  |  |  | |  |  |  |  |  |
|  |  |  | |  |  |  |  |  |
| Skin |  |  | |  |  |  |  |  |
| Dimri, 1995 | Y | 10 | | 40 | x̄ 35.0,  IQR 31.0 - 37.8 | Non cancer tissue; Mohs’ micrographic surgery | SAβ-gal | IHC |
|  | O | 10 | | 60 | x̄ 75.5,  IQR 73.0 - 80.3 |  |  |  |
| Severino, 2000 | **-** | 53 | | 64 | Range 14 - 84 | Non cancer tissue; Mohs’ micrographic surgery | SAβ-gal | IHC |
| El-Domyati, 2003 | **-** | 36 | | 50 | x̄ 35.1,  Range 6 - 77 | Facial and abdominal tissue | p53 | IHC |
| Ressler, 2006 | **-** | 33 | | nr | Range 0 - 95 | Outside disease area | p16 | IHC |
| Lee, 2010 | **-** | 2 | | nr | Neonatal, 86 | Prepuce and abdominal tissue | p16 | IHC |
| Klement, 2012 | **-** | 4 | | nr | Range 18 - 77 | Skin | Ki67 | IF |
| Spandau, 2012 | **-** | 25 | | 79 | Range 22 - 89 | Upper buttock and dorsal forearm | 53BP1 | IHC |
| Dreesen, 2013 | Y | 1 | | nr | 1 | Scalp, eyelid, above upper lip tissue | Lamin B | IHC |
|  | O | 3 | | nr | >60 |  |  |  |
| Marcoux, 2013 | **-** | 10 | | 80 | x̄ 18, Range 13 - 25 | Scalp and buttocks tissue; post chemotherapy | p16, p21 | PCR |
| Waaijer, 2015 | **-** | 178 | | 49 | µ 63.4, SD 6.6 | Upper inner arm | p16 | IHC |
| Lang, 2016 | Y | 9-10 | | nr | Range 18 - 25 | Neck and buttock | miR-15b | qPCR |
|  | O | 9-10 | | nr | Range 60 - 66 |  |  |  |
| Nassour, 2016 | Y | 2 | | 50 | Range 29 - 38 | Skin biopsy | p16, 53BP1 | IHC |
|  | O | 2 | | 100 | Range 65 - 89 |  |  |  |
| Testis |  |  | |  |  |  |  |  |
| Bartkova, 2011 | **-** | 33 | | 100 | Range: Fetal - adult | Testicular tissue outside disease site; orchidectomy | γH2AX | IHC |
| Thymus |  |  | |  |  |  |  |  |
| Kanavaros, 2001 | **-** | 20 | | nr | Range: Fetal - adult | Biopsy no thymic pathology; surgery | Ki67, p16 | IHC |
| Several Tissues |  |  | |  |  |  |  |  |
| Nielsen^A^, 1999 | **-** | nr | | nr | nr | Obtained via surgery or PM | p16 | IHC |
| Nuciforo^B^, 2007 | **-** | nr | | nr | nr | Normal and tumor regions | DDR (γH2AX, ATM, 53BP1, CHK2, p53) | IHC |
| Abbreviations C: Control. CLO: Community Living Older person. CT: Centenarian. D: Disease tissue. DDR: DNA damage response. E: Exploratory Cohort. FC: Flow cytometry. G: Group. IF: Immunofluorescence. IHC: Immunohistochemistry. IQR: Interquartile range. M: Middle age group. nr: Not reported. O: Old age group. OCT: Offspring centenarian. OS: Older age group sedentary lifestyle. OT: Older age group training lifestyle. PCR: Polymerase chain reaction. PM: Post-mortem. SCT: Super centenarian. SD: Standard deviation. SE: Standard error. V: Validation cohort. WB: Western blot. Y: Young age group. µ: Mean. x̄: Median.  ^A^: Tissue types: brain, spinal cord/peripheral nerves, heart, lung, liver, spleen, kidney, bladder, uterus, ovary, breast, testis, epididymis, prostate, oesophagus, stomach, intestines, pancreas, pituitary, adrenal, thyroid, parathyroid and salivary glands, skin, tonsil, lymph node and bone marrow.  ^B^: Mix of normal and tumour tissue. Tissue types: breast, lung, colon-rectum, kidney, larynx, stomach, hematopoietic system, skin, soft tissue, bone. | | | | | | | | |

| Supplementary Table 2. Qualitative association of the magnitude of senescence with chronological age. | | | | |  |
| --- | --- | --- | --- | --- | --- |
| Author, Year | **No. Assoc** | **Assoc Age** | **Specification** | **Note** | |
| Adipose |  |  |  |  | |
| Justice, 2017 | 1 | ± | r: 0.31 p16 % positive |  | |
| Xu, 2015 | 2 | +, + | ΔOY: 7% senescent cells γH2Ax & Ki67 |  | |
| Artery |  |  |  |  | |
| Marchland, 2011 | 2 | ±, ± | FC O/M: 1.1 p21 & cyclin D |  | |
| Morgan, 2013 | 4 | +*,+,+*,+* | FC O/Y: 1.9, p21; 1.7, IL-6; 7.5, IL-8; 2.1, MCP-1 |  | |
| Rossman, 2017 | 3 | +,+,+ | Δ OS/Y: 0.5, p53, p21, p16  Δ OS/OE: 0.4, p53 and p16; 0.2, p21 |  | |
| Blood |  |  |  |  | |
| Schonland, 2003 | 1 | nr | β: 0.017 % Ki67 positive/year |  | |
| Liu, 2009 | 1 | +* | r^2^: 0.40 p16 (log^2^) mRNA |  | |
| Werner, 2011 | 1 | +* | FC O/Y: 2.16 p53 |  | |
| Sanoff a, 2014 | 1 | +* | β: 0.06 p16 (log^2^) per year |  | |
| Sanoff b, 2014 | 1 | ± | ΔOY: -0.23 p16 (log^2^) |  | |
| Pustavoitau, 2015 | 1 | + | r: 0.38 p16 (log^2^) per year |  | |
| Brain |  |  |  |  | |
| Bhat, 2012 | 1 | +* | ΔOY: 43% p16 positive |  | |
| Kang, 2016 | 3 | + | ΔOY:75 AFU p16 Oligodendrocytes,  ΔOY:40 AFU p16  Pyramidal Neurons,  ΔOY: 43 AFU p16 Astrocytes |  | |
| Eye |  |  |  |  | |
| Song, 2008 | 3 | +*,±,± | ΔOY: 0.37 rel.exp. p16  ΔOY: -0.54 rel.exp. p21  ΔOY: 0.06 rel.exp. p53 |  | |
| Abdouh, 2012 | 1 | +* | ΔOY: 0.56 rel.exp. bmi1 | a | |
| Zang, 2012 | 1 | +* | r: 0.56 rel. exp. p16 |  | |
| Cao, 2013 | 1 | +* | ΔOY: 0.83 higher stain  IL-1RA |  | |
| Gastro-intestinal |  |  |  |  | |
| Going, 2002 | 1 | nr | *‘NS SAβ-gal activity and age’* | b | |
| Helman, 2016 | 2 | +,+ | r^2^=0.83 rel. exp. p16  14 Yr 2%, 48 Yr 40% pos SA-βgal |  | |
| Dock, 2017 | 4 | -,-, | CD8^+^Ki-67^+^ (p=0.0093), Gut Mucosa cells: CD8^+^Ki-67^+^ (NS), |  | |
| Heart |  |  |  |  | |
| Kajstura, 2010 | 1 | +* | r: 0.83 p16 % positive |  | |
| Kidney |  |  |  |  | |
| Chkhotua, 2003 | 6 | +,+*,+,+*,+,± | r: 0.30-0.73 p16 % positive | c | |
| Melk, 2004 | 6 | +*,±, ±,−*, ±,± | Cortex: β: 0.016 rel. exp. p16  Β: 0.0003 rel. exp. p53  Β: 0.003 rel. exp. p21  Β: -0.013 rel. exp. MMP1  Β: 0.002 rel. exp TGFβ1  Medulla: r^2^: 0.03 rel. exp. p16 |  | |
| Sis, 2007 | 4 | +*,±,±,+ | r^2^: 0.04-0.07 % p16 positive | c,d | |
| Koppelstaetter, 2008 | 2 | ±,+* | r: -0.01 rel. exp. p21  r: 0.30 rel. exp. p16 |  | |
| Verzola, 2008 | 3 | ±,±,± | Glomerulus r: -0.02 p16 % positive Tubule r: 0.02 p16 % positive  Tubule r: 0.38 SAβ-gal % positive |  | |
| McGlynn, 2009 | 1 | +* | r: 0.33 rel. exp. p16 |  | |
| Liu, 2012 | 3 | ±,±,± | r: 0.12 SAβ-gal % positive  r: 0.25 p16 % positive  r: 0.11 p21 % positive |  | |
| Gingell-Littlejohn, 2013 | 1 | +* | r: 0.60 rel. exp. p16 |  | |
| Berkenkamp, 2014 | 1 | +* | r: 0.27 cyclin d % positive |  | |
| Gunther, 2017 | 1 | + | r^2^: 0.18 CDKN2A |  | |
| Lung |  |  |  |  | |
| Shivshankar, 2011 | 2 | +*,+* | ΔOY: 2.27 rel. exp. p16  ΔOY: 0.41 rel. exp. mH2A |  | |
| Disayabutr, 2016 | 1 | ± | ‘*age was not a predictor for SA-βgal activity’* |  | |
| Prostate |  |  |  |  | |
| Choi, 2000 | 1 | ± | Mean age SAβ-gal negative tissue 62.0 (SD 7.1)  Mean age SAβ-gal positive tissue 60.4 (SD 5.9) |  | |
| Castro, 2003 | 1 | nr | ΔOY: 0.31 SAβ-gal activity |  | |
| Skin |  |  |  |  | |
| Dimri, 1995 | 2 | nr;nr | SAβ-gal pos per biopsy:  Epidermis: Y: 4-, 3 ±, 2 +,  1 ++  O: 2 ±, 2++, 6 +++  Dermis Y: 10 -  O: 1 -, 4 ++, 5 +++ |  | |
| Severino, 2000 | 1 | ± | r: 0.09 SAβ-gal % positive |  | |
| El-Domyati, 2003 | 2 | +*,± | β: 0.06 p53 intensity/positive facial  β: 0.001 intensity/positive abdominal |  | |
| Ressler, 2006 | 1 | +* | ΔOY: 6 no. p16 positive |  | |
| Lee, 2010 | 1 | nr | *"P16 stained cells were not detected well in Age 1 and Age 10 and numerous p16 stained cells were observed in Age 86"* |  | |
| Klement, 2012 | 1 | +* | ΔOY: 13.2 Ki67 % positive | a | |
| Spandau, 2012 | 1 | +* | ΔOY: 22.353BP1 % positive |  | |
| Dreesen, 2013 | 2 | nr;nr | *"Robust levels of LMNB1 .. in the majority of young epidermal kerationocytes" " .. in skin from old donors, LMNB1.. were reduced" ".. the number of Ki-67–positive cells in the basal layer was dramatically reduced in aged skin"* | a | |
| Marcoux, 2013 | 2 | ±,± | r: -0.39 rel. exp. p16  r: -0.52 rel. exp. p21 |  | |
| Waaijer, 2015 | 2 | +,± | Tertiles p16 % positive cells were positively associated with age in epidermal samples not dermal samples. |  | |
| Lang, 2016 | 1 | ± | *miR-15b expression lower in older individuals* |  | |
| Nassour, 2016 | 1 | + | ΔOY: 8% p16 positive |  | |
| Testis |  |  |  |  | |
| Bartkova, 2011 | 1 | nr | *Adult testes: ".. strong staining for γH2AX in spermatocytes.." Foetal testes: "..evident γH2AX staining that is characterized by numerous fine foci"* | b | |
| Thymus |  |  |  |  | |
| Kanavaros, 2001 | 2 | +*,nr | ΔOY: 62.6% Ki67 positive; *increase p16 positive cells with age* | a,b | |
| Several Tissues |  |  |  |  | |
| Nielsen, 1999 | Several | nr | *".. p16 expression in newborns was present only in Hassall's corpuscles, scattered thymic lymphocytes, and rare epithelial cells of the pancreas."* | e | |
| Nuciforo, 2007 | Several | ± | *" .. there is no significant association between individual activated DDR markers and age, .."* | b,e | |
| Abbreviations: AU: Arbitary Units. FC: Fold change. MFI: Mean fluorescence intensity. M: Middle age group. No. Assoc: number of associations. nr: Not reported. O: Old age group. r: correlation coefficient. R^2^: coefficient of determination, Rel. exp.: Relative expression. Y: young age group. β: Slope, Δ: difference, +*: positive significant association (p<0.05). +: positive trend (0.05<p<0.10), ±: inconclusive (p>0.10 or termed ‘ns’ in source report). −: negative trend (0.05<p<0.10). −*: negative significant association (p<0.05). | | | | |  |

**Supplementary Figures**

**Supplementary Figure 1A.** Correlation Meta-Analysis between senescence marker and age for tissue from adipose and artery.


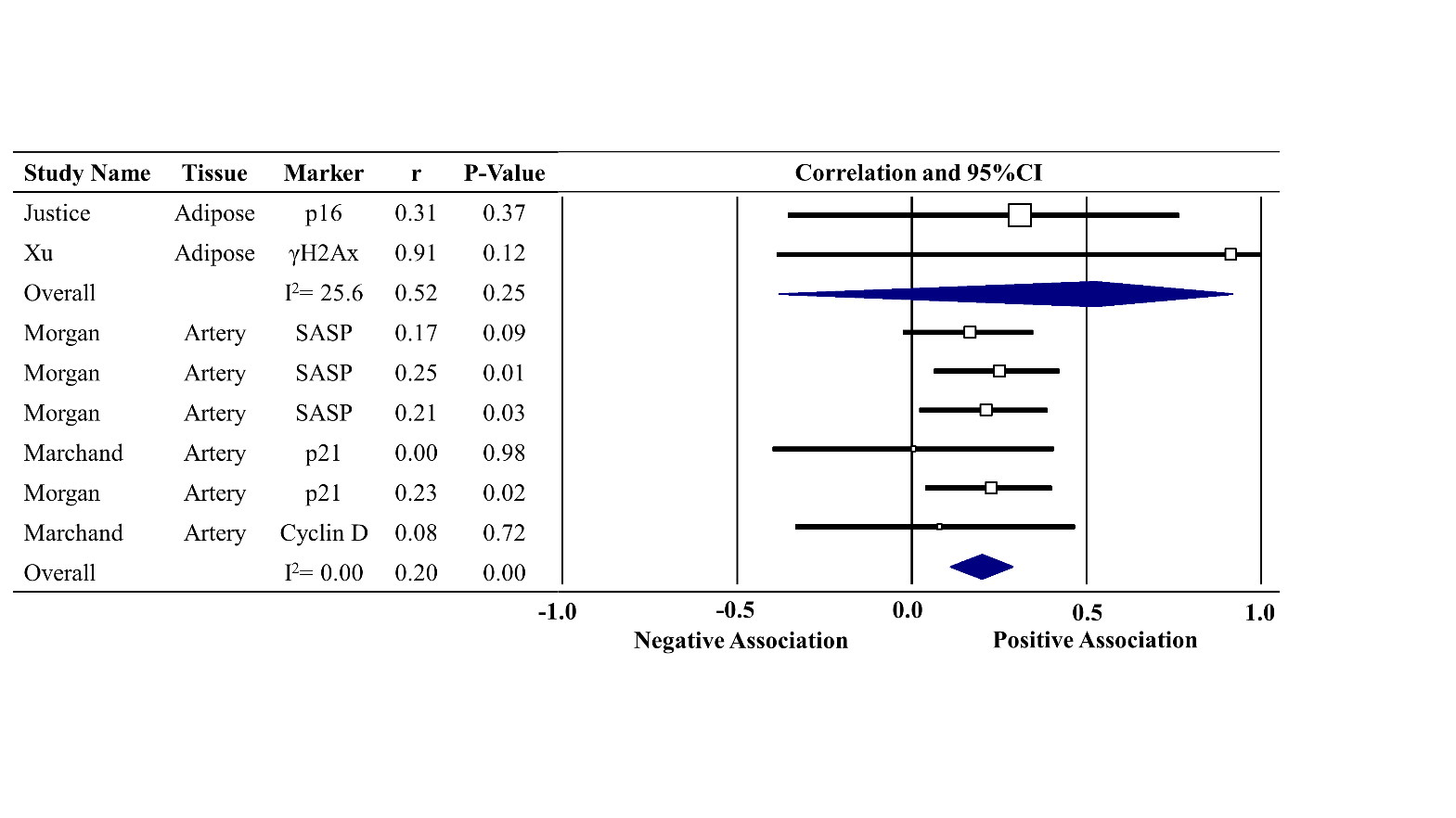


**Supplementary Figure 1B.** Correlation Meta-Analysis between senescence marker and age for tissue from the blood.


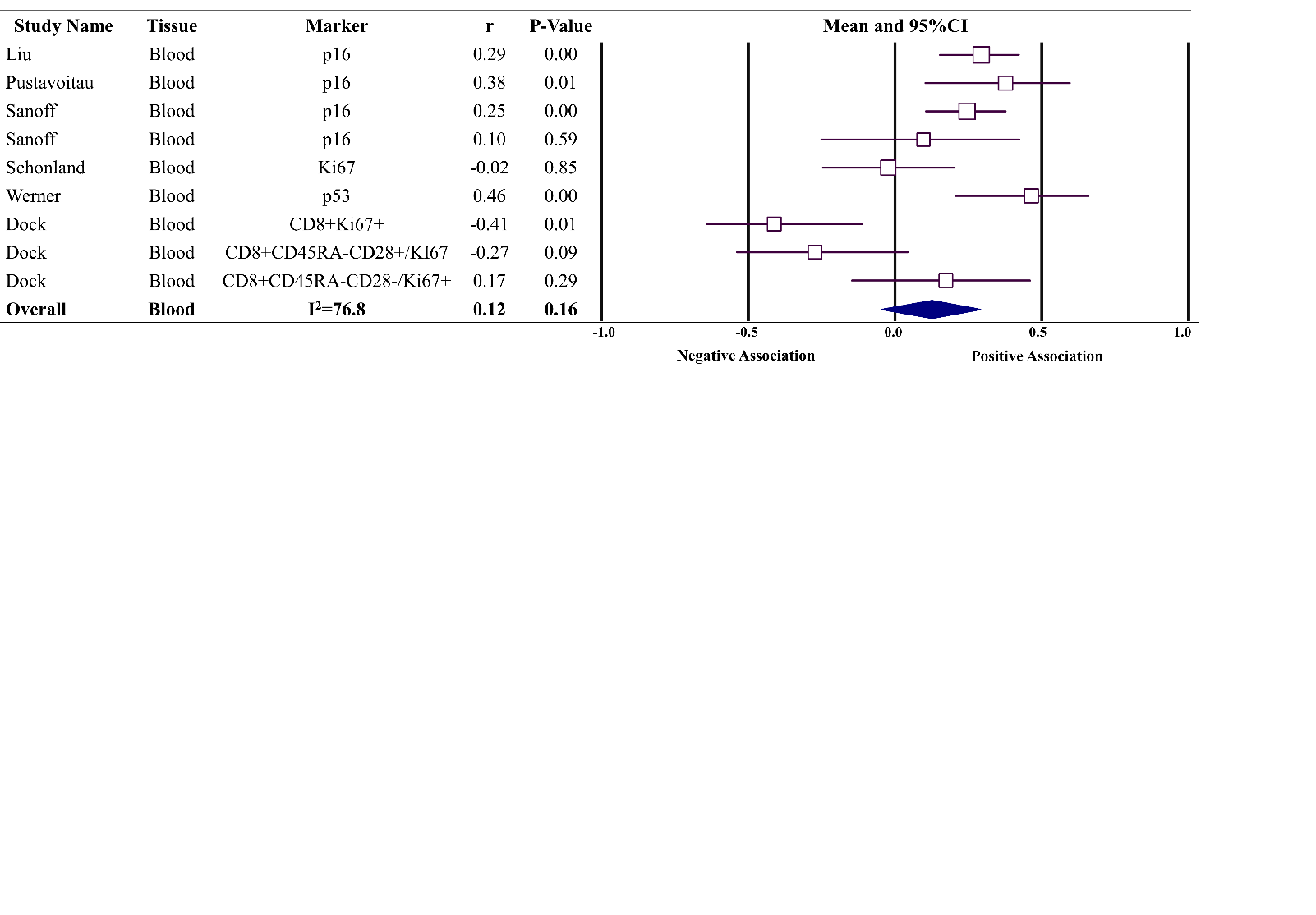


**Supplementary Figure 1C.** Correlation Meta-Analysis between senescence marker and age for tissue from the brain, eye and gut.


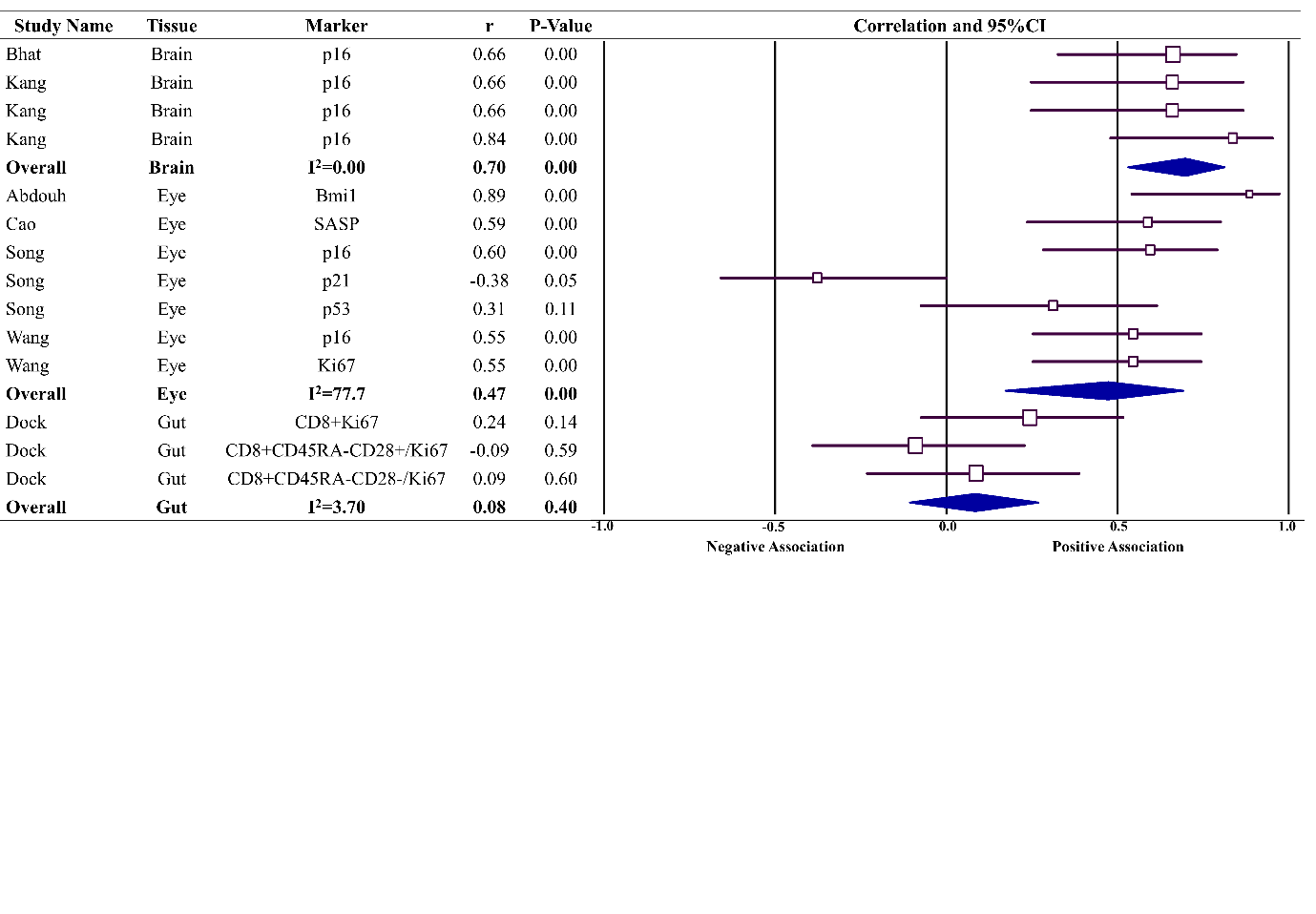


**Supplementary Figure 1D.** Correlation Meta-Analysis between senescence marker and age for tissue from the kidney.


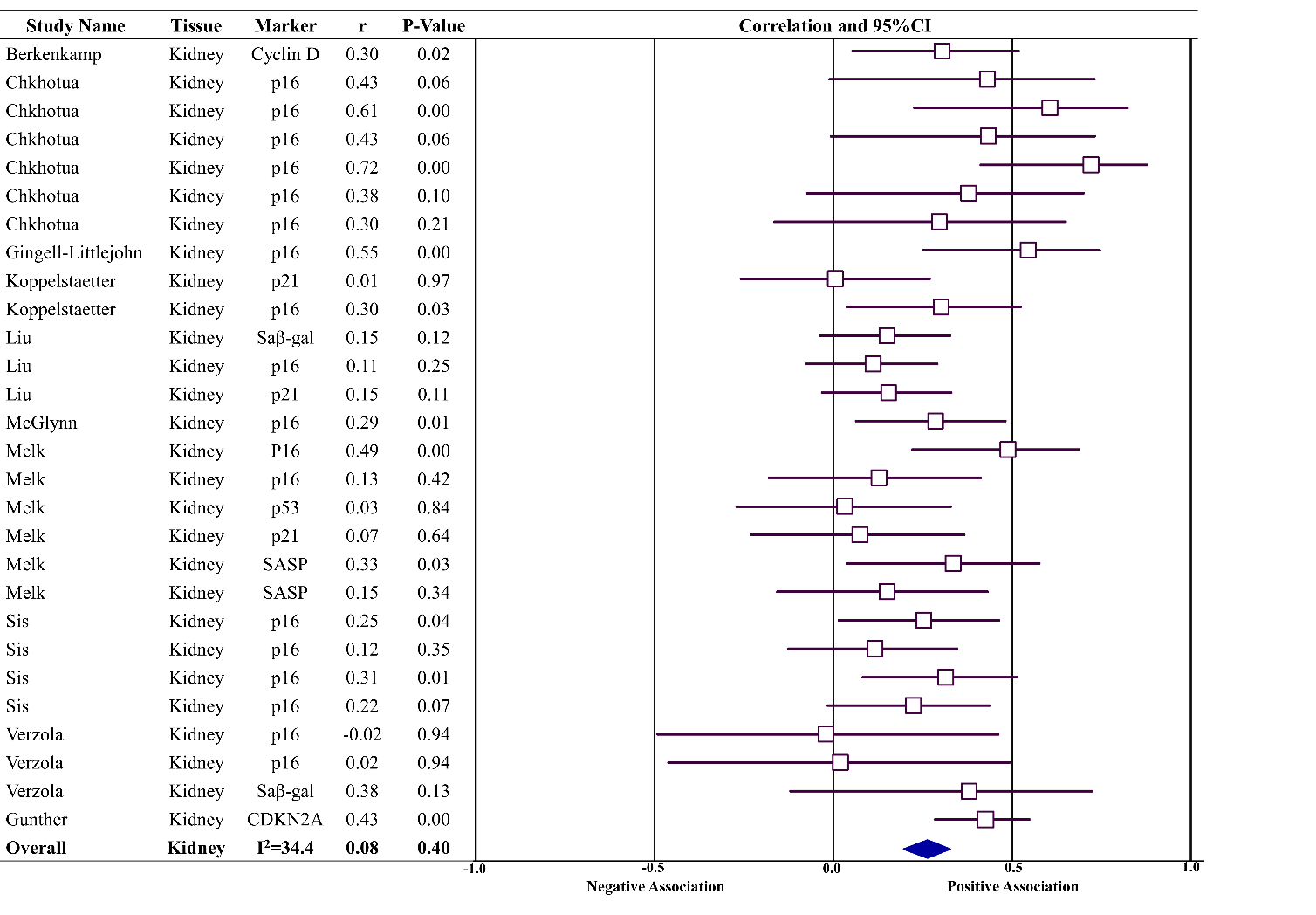


**Supplementary Figure 1E.** Correlation Meta-Analysis between senescence marker and age for tissue from the heart, lung, pancreas and prostate.

**
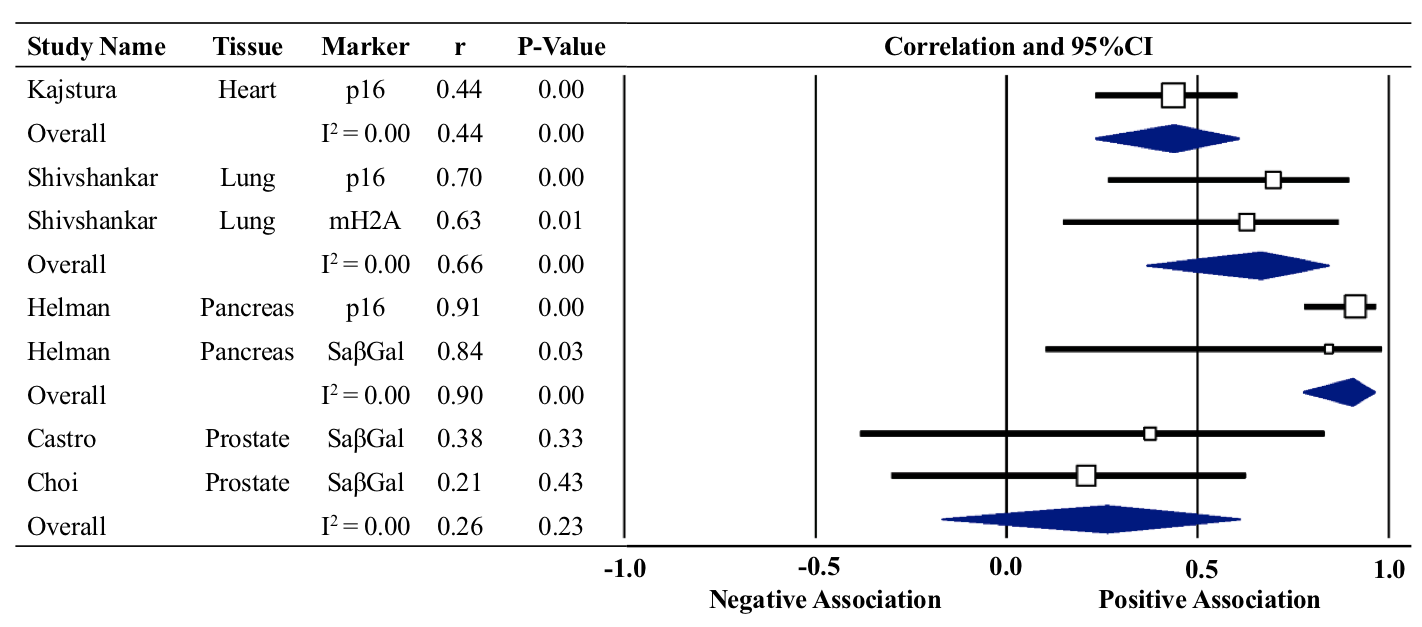
**

**Supplementary Figure 1F.** Correlation Meta-Analysis between senescence marker and age for tissue from the skin.


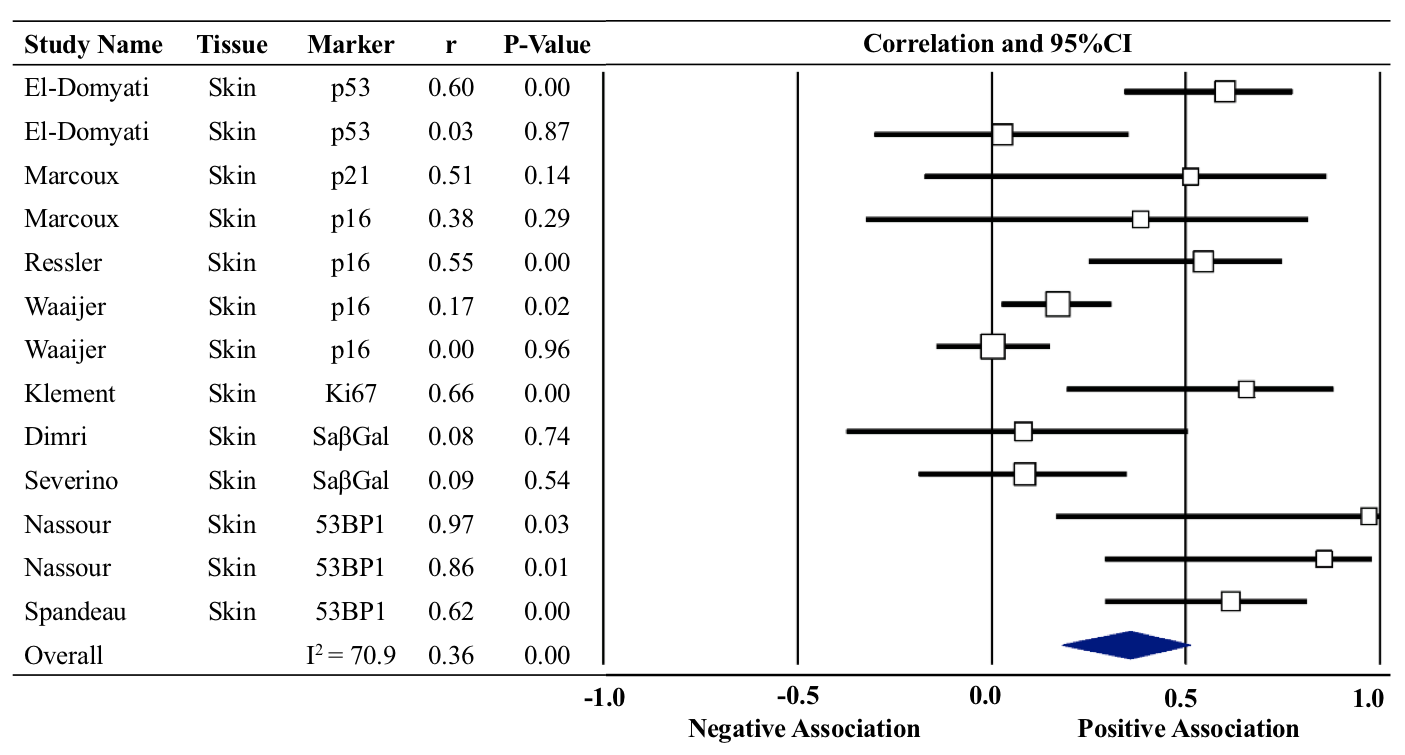


**Supplementary Figure 1G.** Correlation Meta-Analysis between senescence marker and age for tissue from the thymus and vein.

**
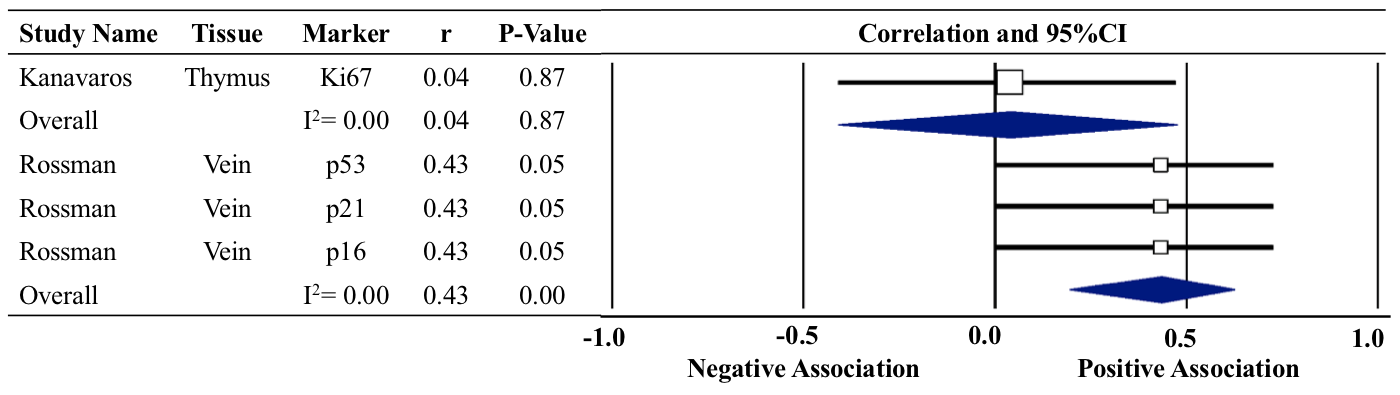
**

**Supplementary Figure 2A** Correlation Meta-Analysis between senescence marker and age for Saβ-Gal.

**
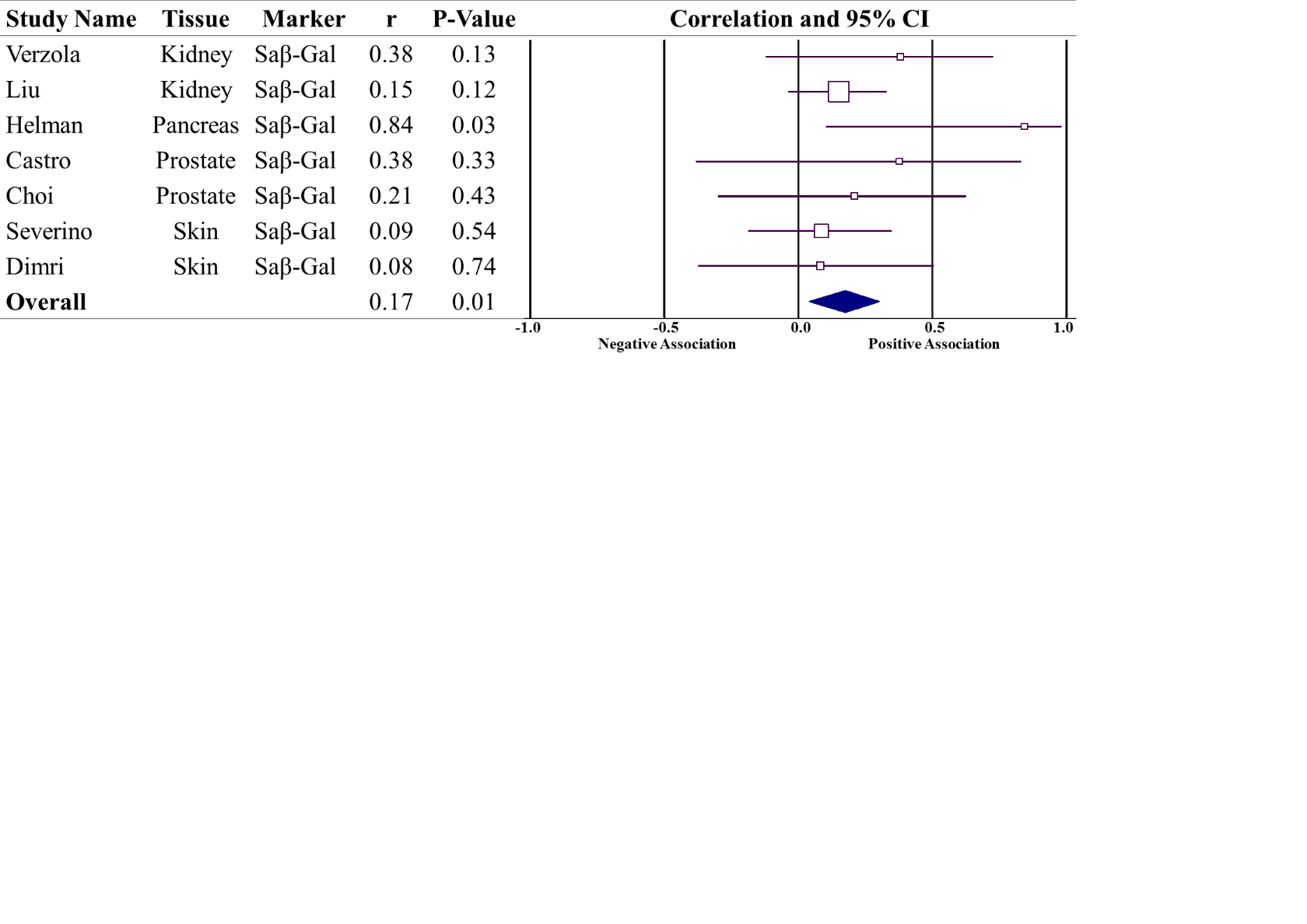
.**

**Supplementary Figure 2B** Correlation Meta-Analysis between senescence marker and age for p16^Ink4a^ (Cell Cycle Regulator)

**
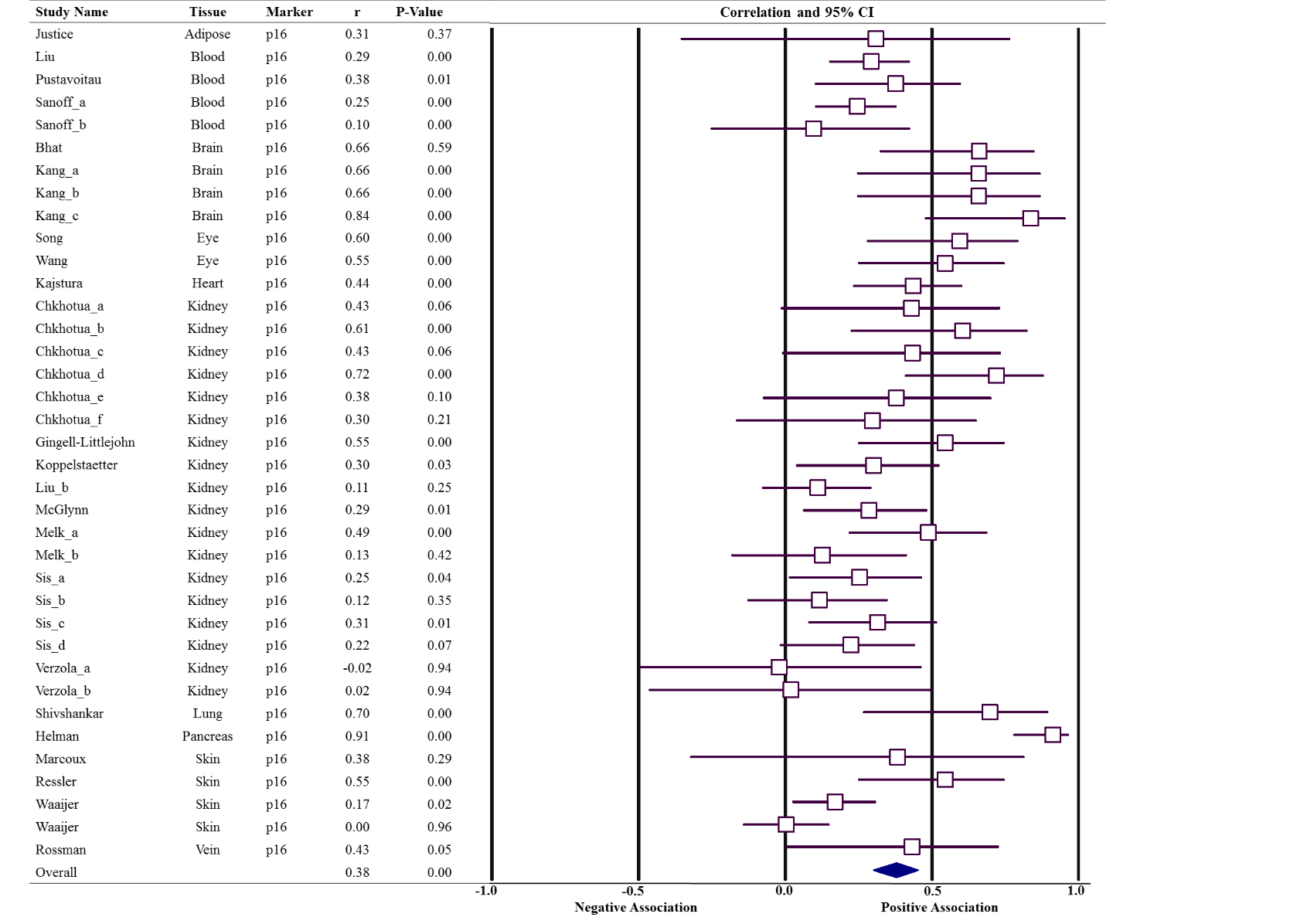
**

**Supplementary Figure 2C** Correlation Meta-Analysis between senescence marker and age for p21 (Cell Cycle Regulator).


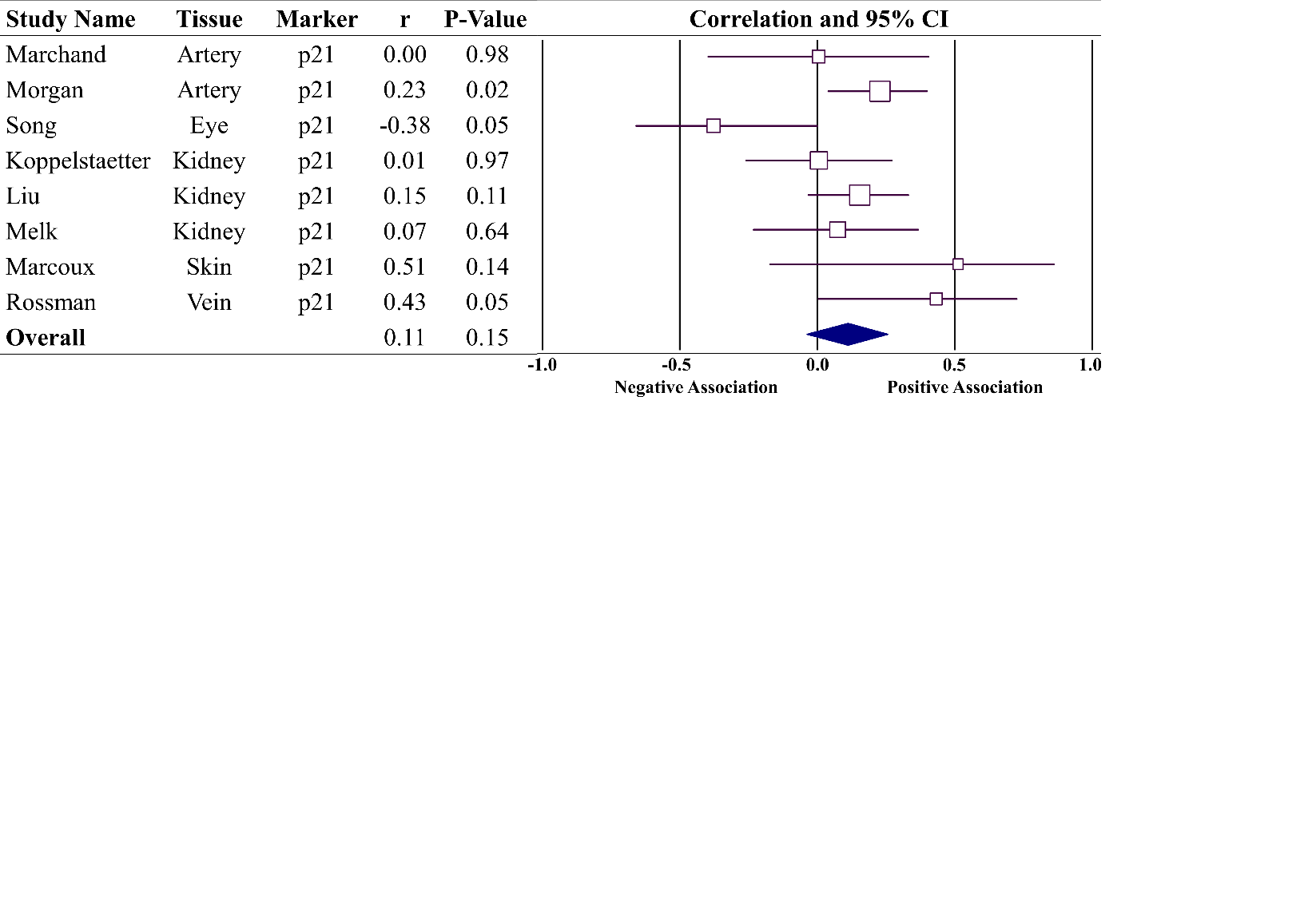


**Supplementary Figure 2D** Correlation Meta-Analysis between senescence marker and age for p53 (Cell Cycle Regulator).


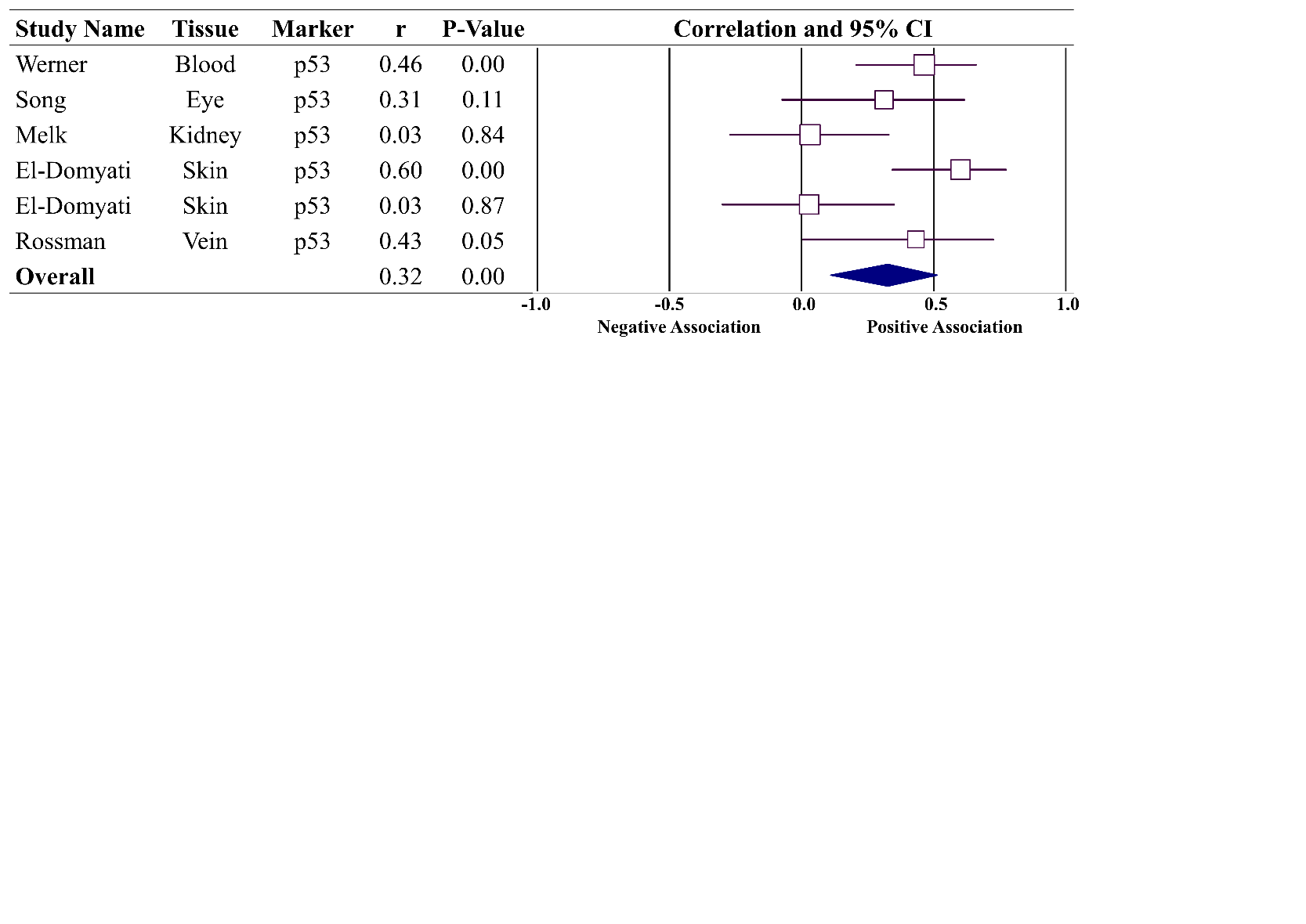


**Supplementary Figure 2E** Correlation Meta-Analysis between senescence marker and age for other cell cycle regulators


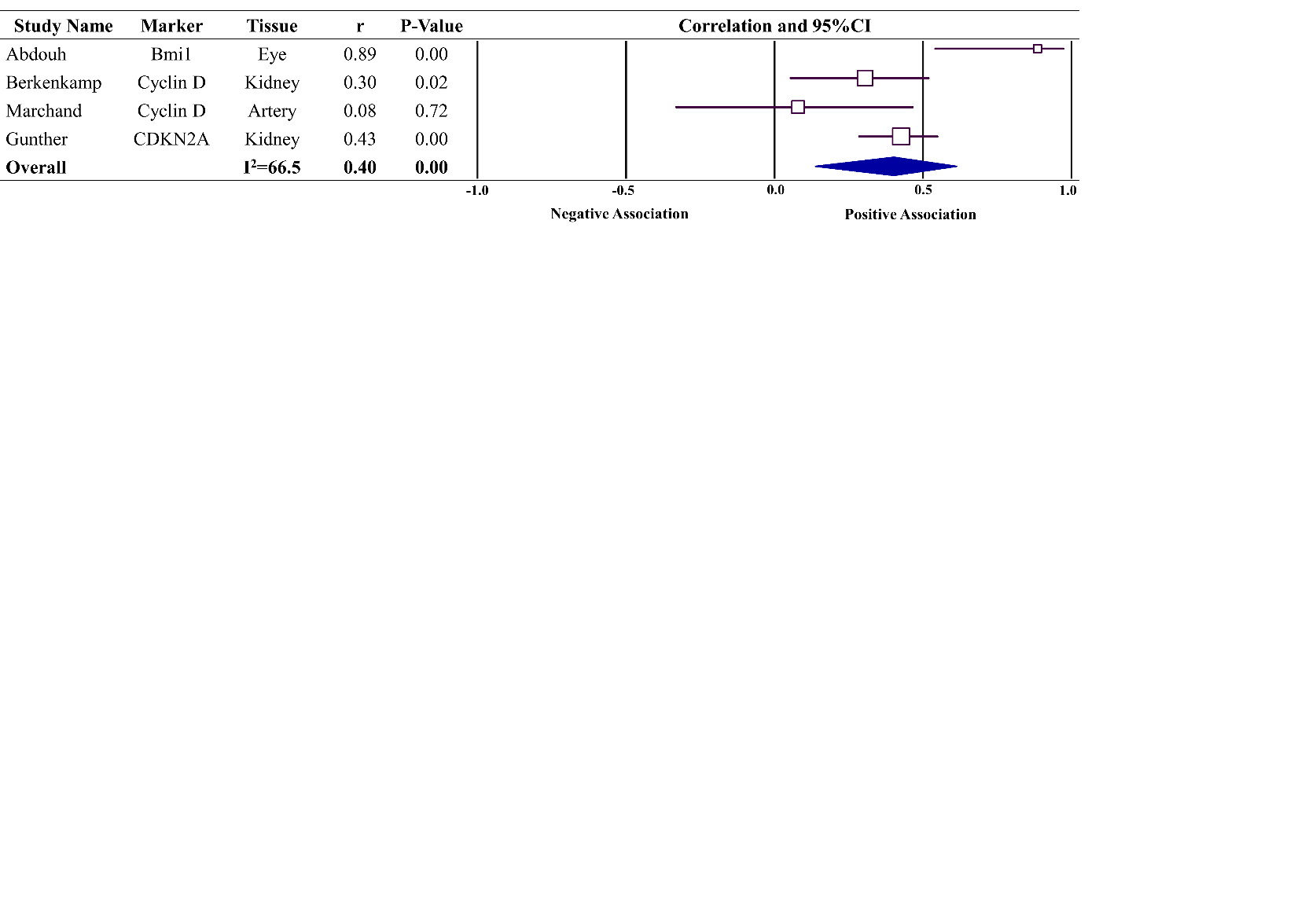


**Supplementary Figure 2F** Correlation Meta-Analysis between senescence marker and age for DNA Damage markers.


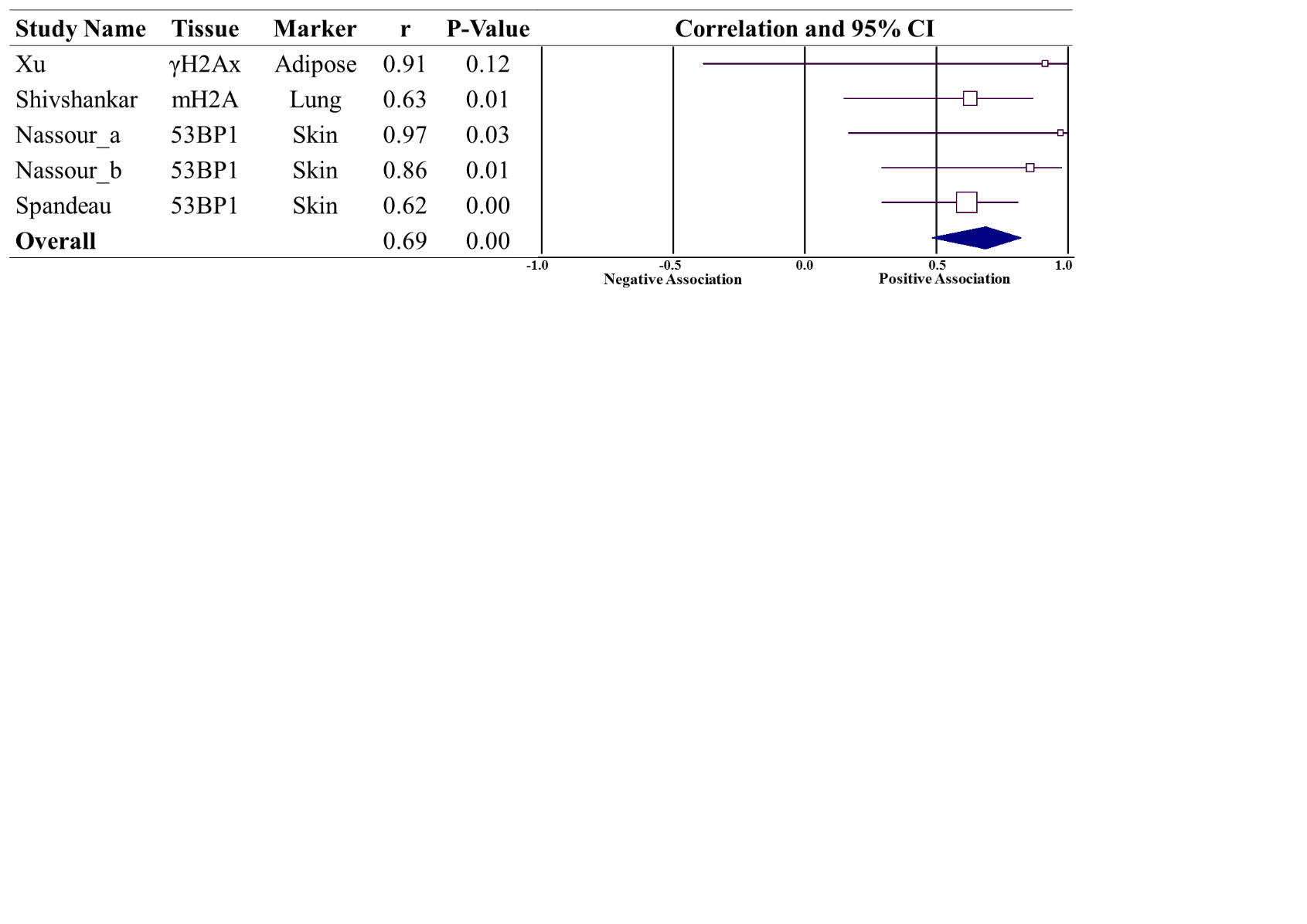


**Supplementary Figure 2G** Correlation Meta-Analysis between senescence marker and age for proliferation

**
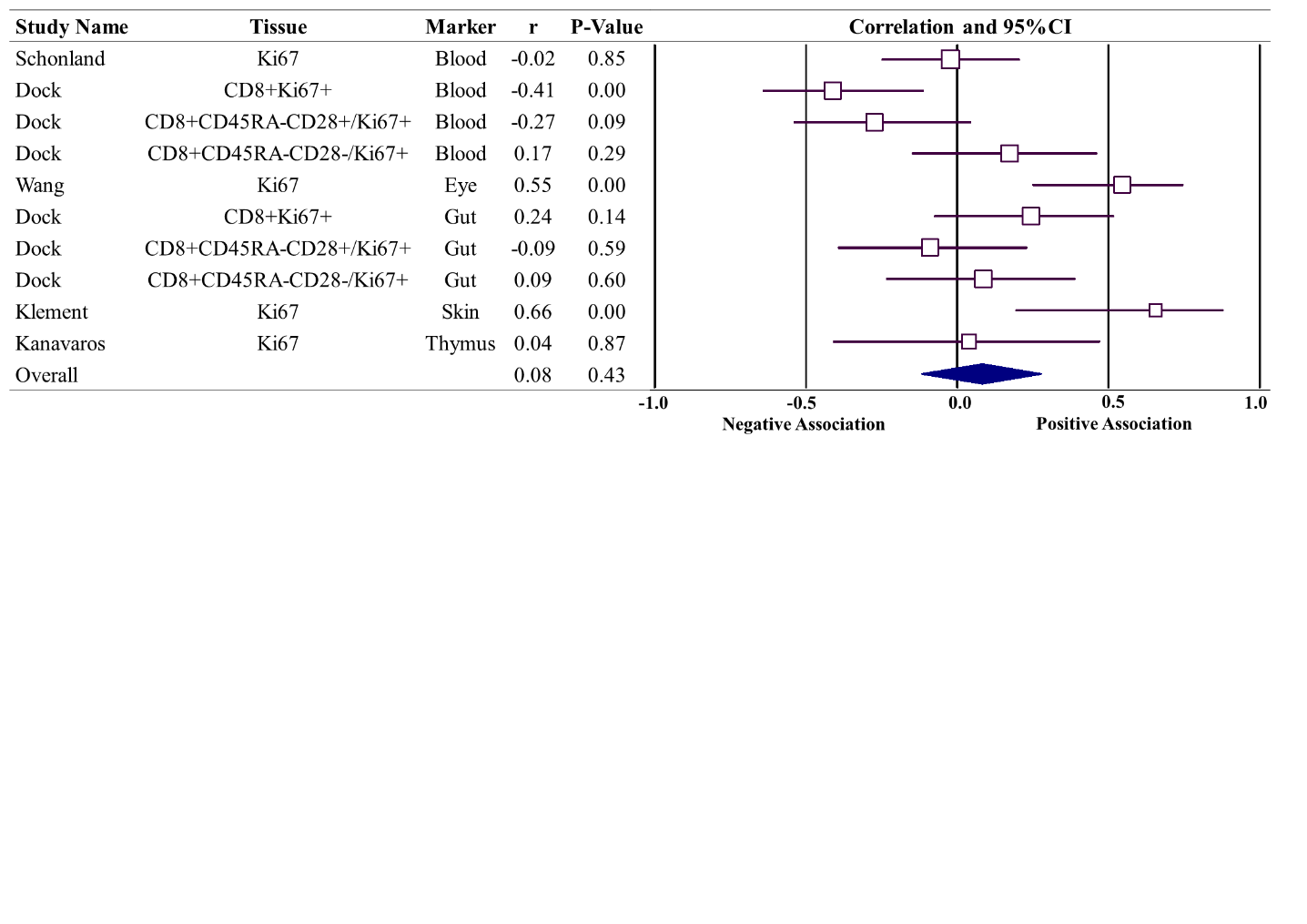
**

**Supplementary Figure 2H** Correlation Meta-Analysis between senescence marker and age for markers of the senescence associated secretory phenotype (SASP).


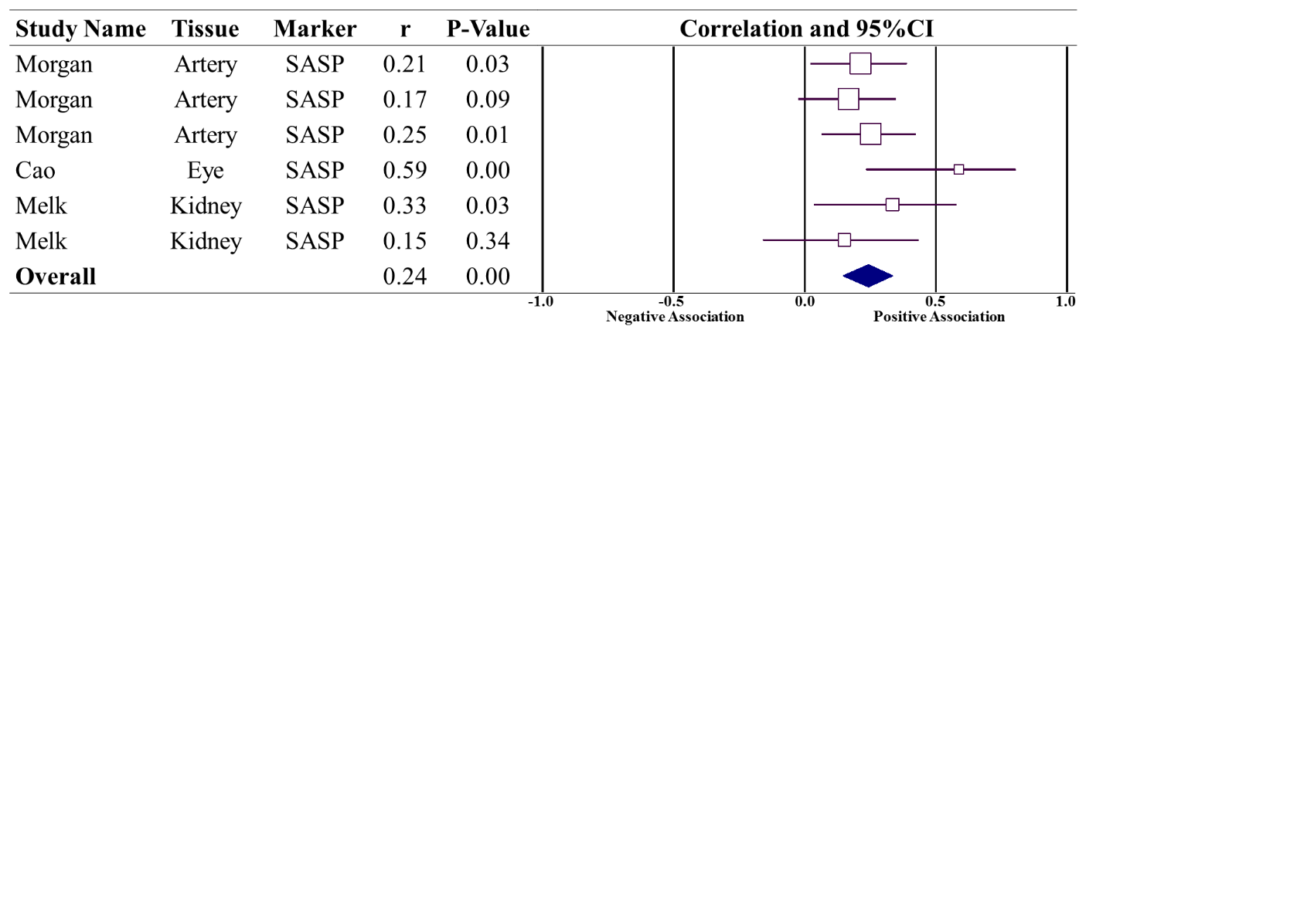


**Supplementary Figure 3A** Meta-Analysis for change in slope/10 years for senescence marker and age sub-grouped by tissue - adipose and blood.


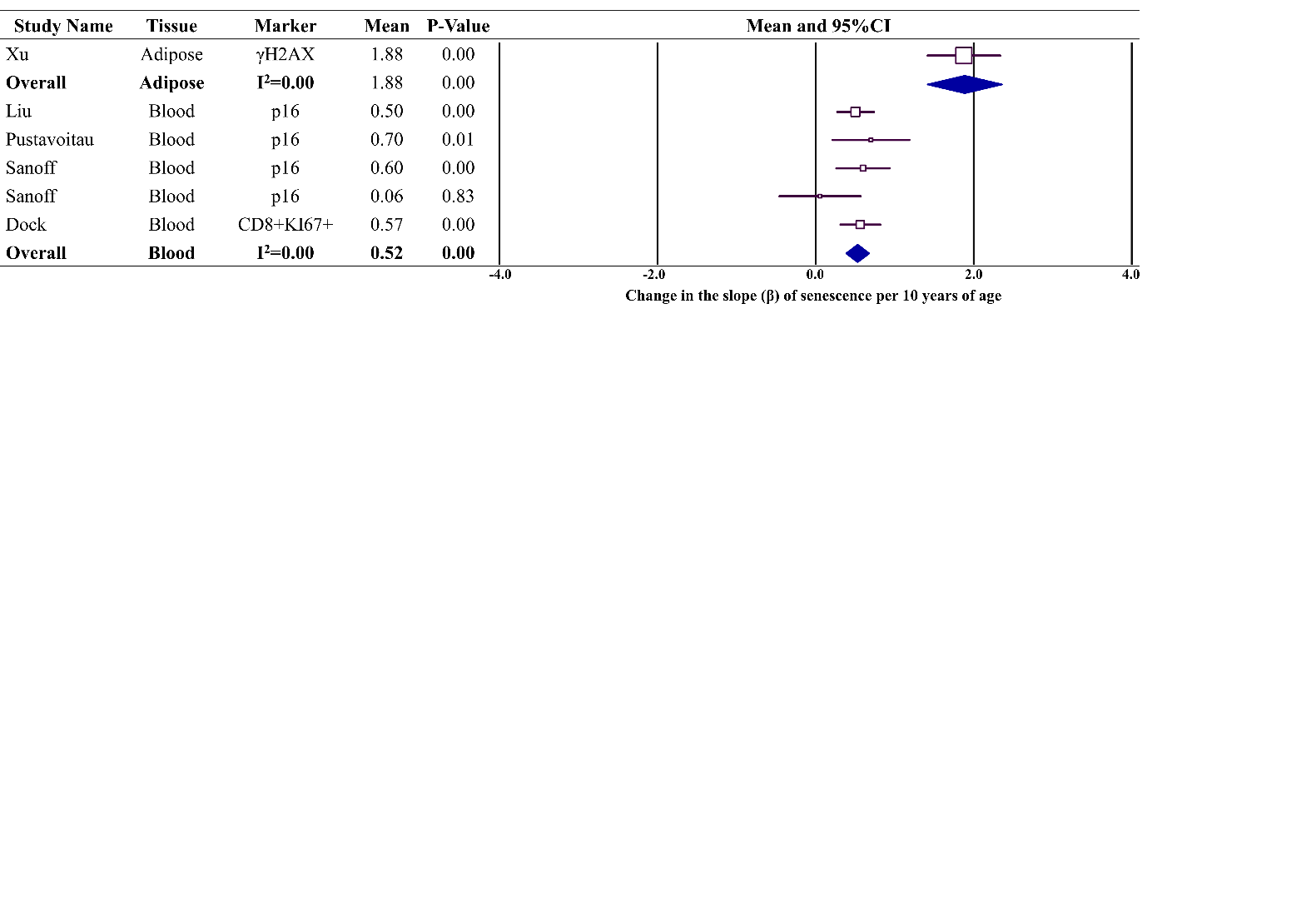


**Supplementary Figure 3B** Meta-Analysis for change in slope/10 years for senescence marker and age sub-grouped by tissue - brain and eye.


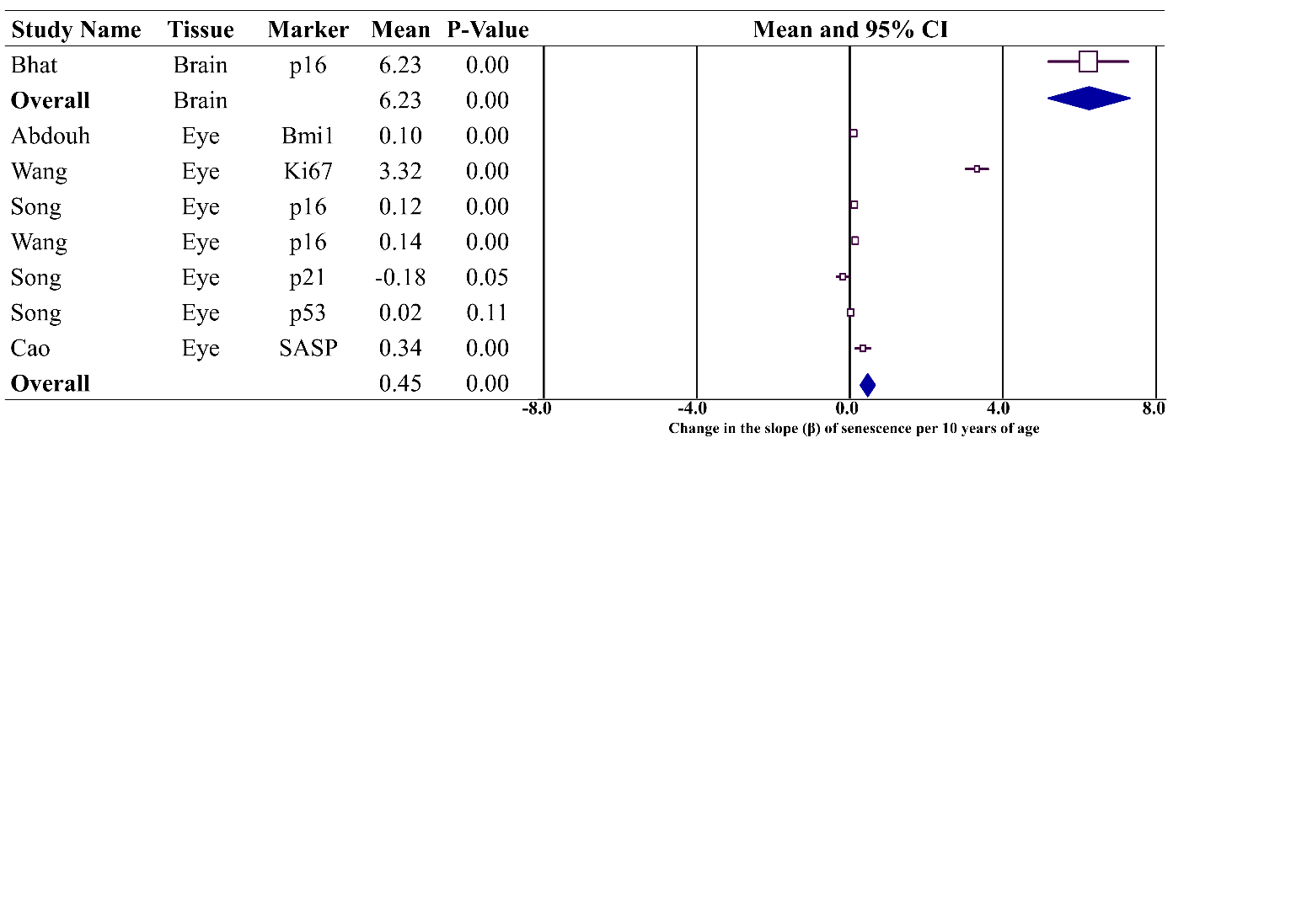


**Supplementary Figure 3C** Meta-Analysis for change in slope/10 years for senescence marker and age sub-grouped by tissue - gut and heart.


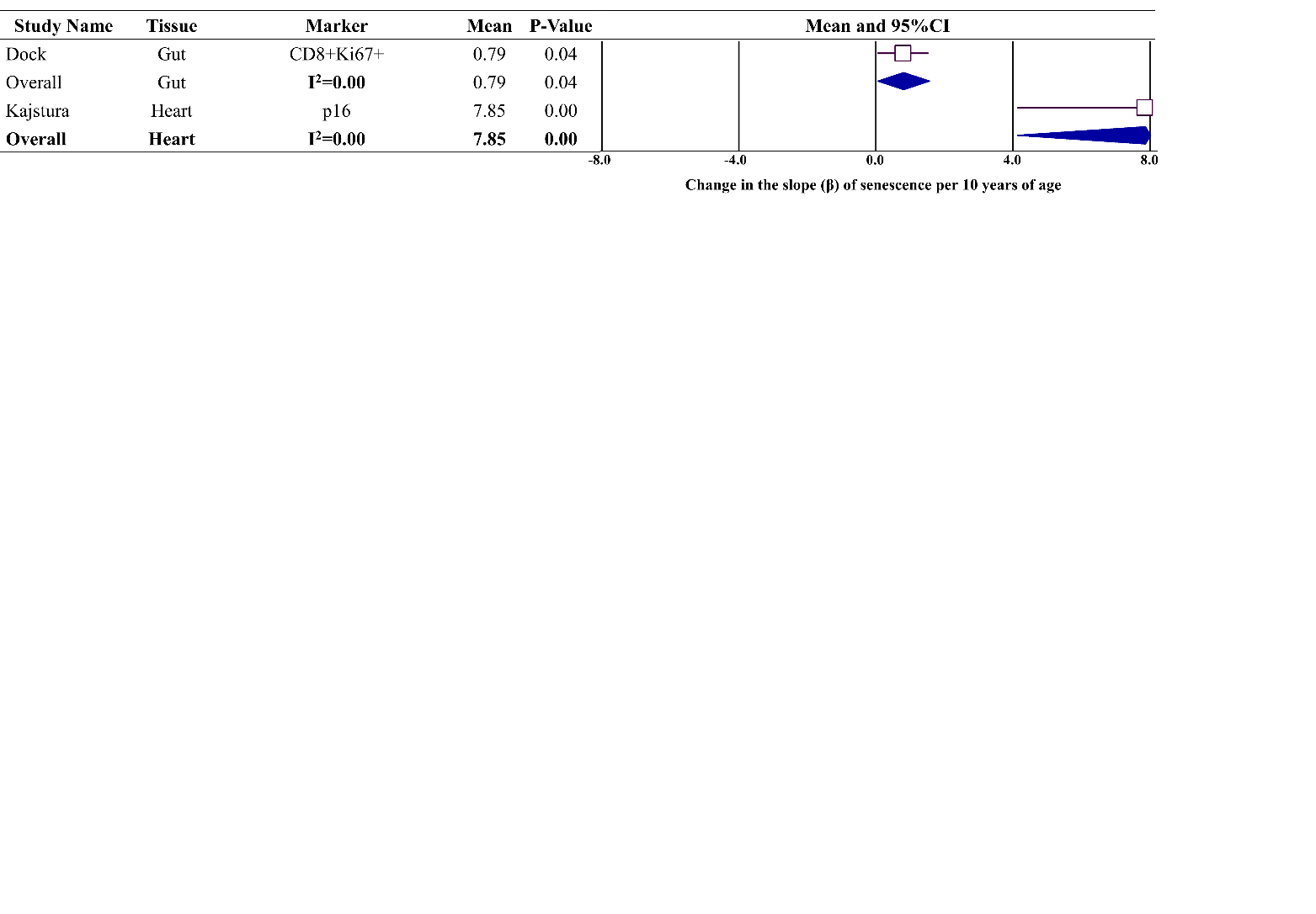


**Supplementary Figure 3D** Meta-Analysis for change in slope/10 years for senescence marker and age sub-grouped by tissue - kidney


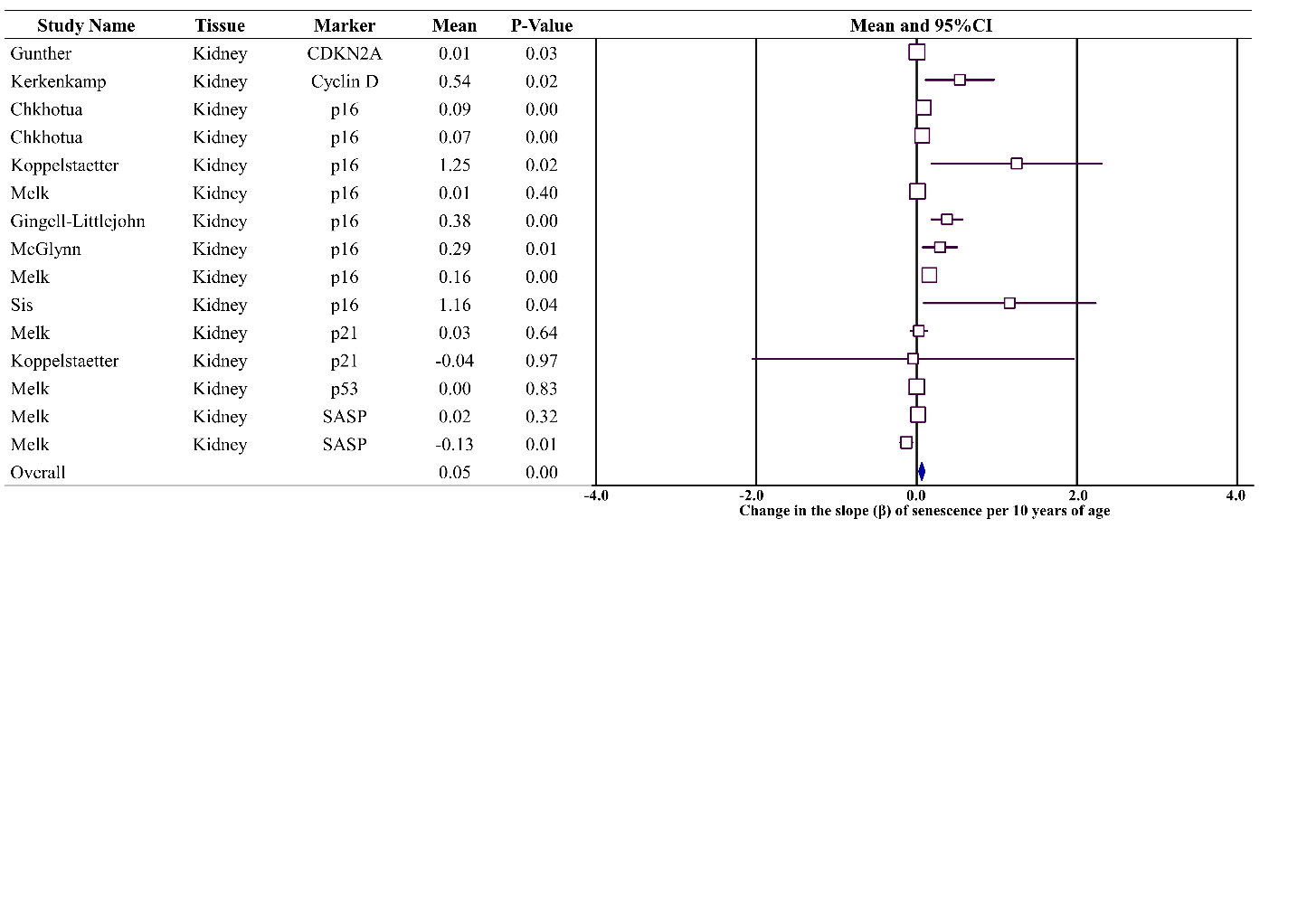


**Supplementary Figure 3E** Meta-Analysis for change in slope/10 years for senescence marker and age sub-grouped by tissue – lung, pancreas and prostate.


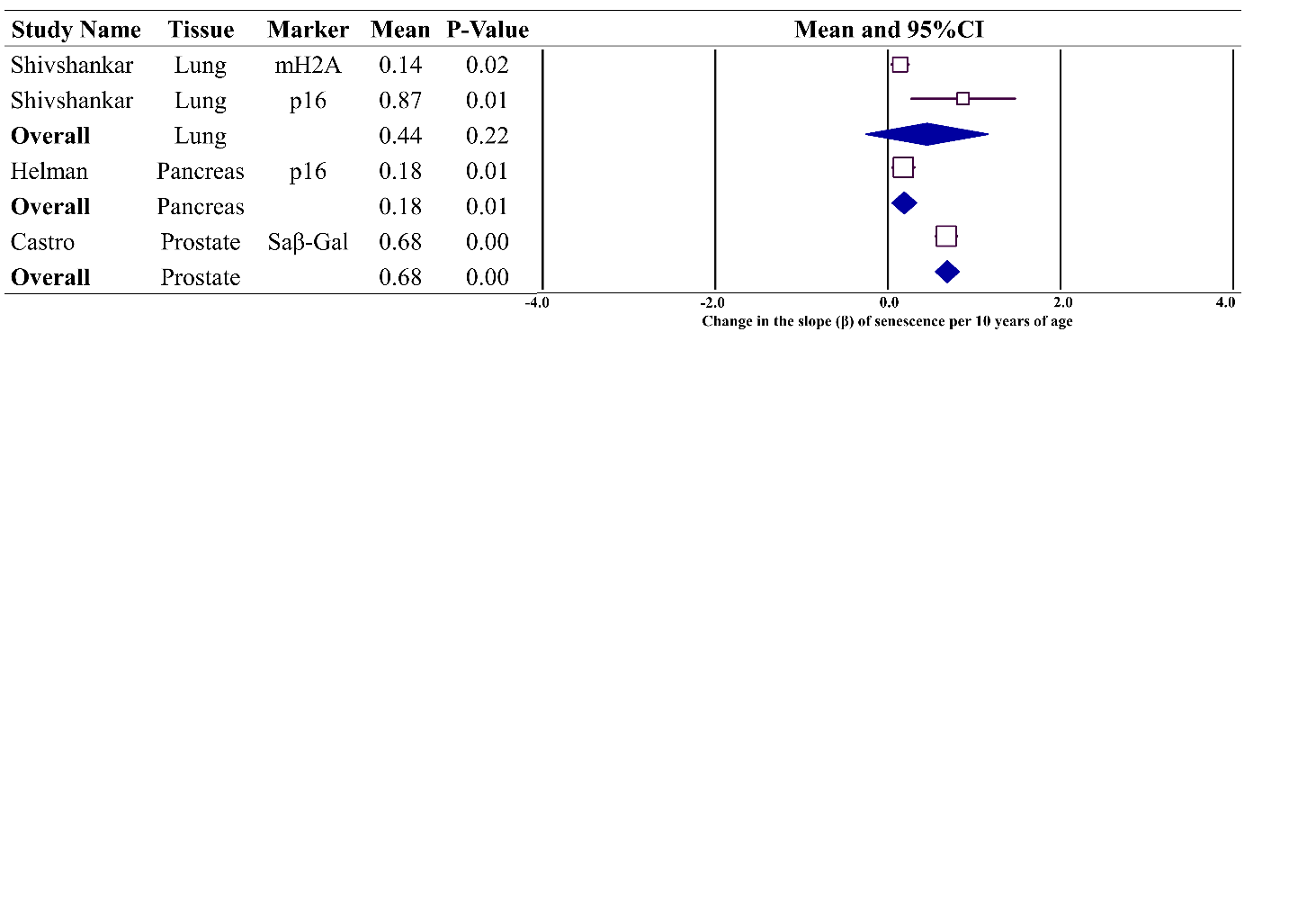


**Supplementary Figure 3F** Meta-Analysis for change in slope/10 years for senescence marker and age sub-grouped by tissue – skin.


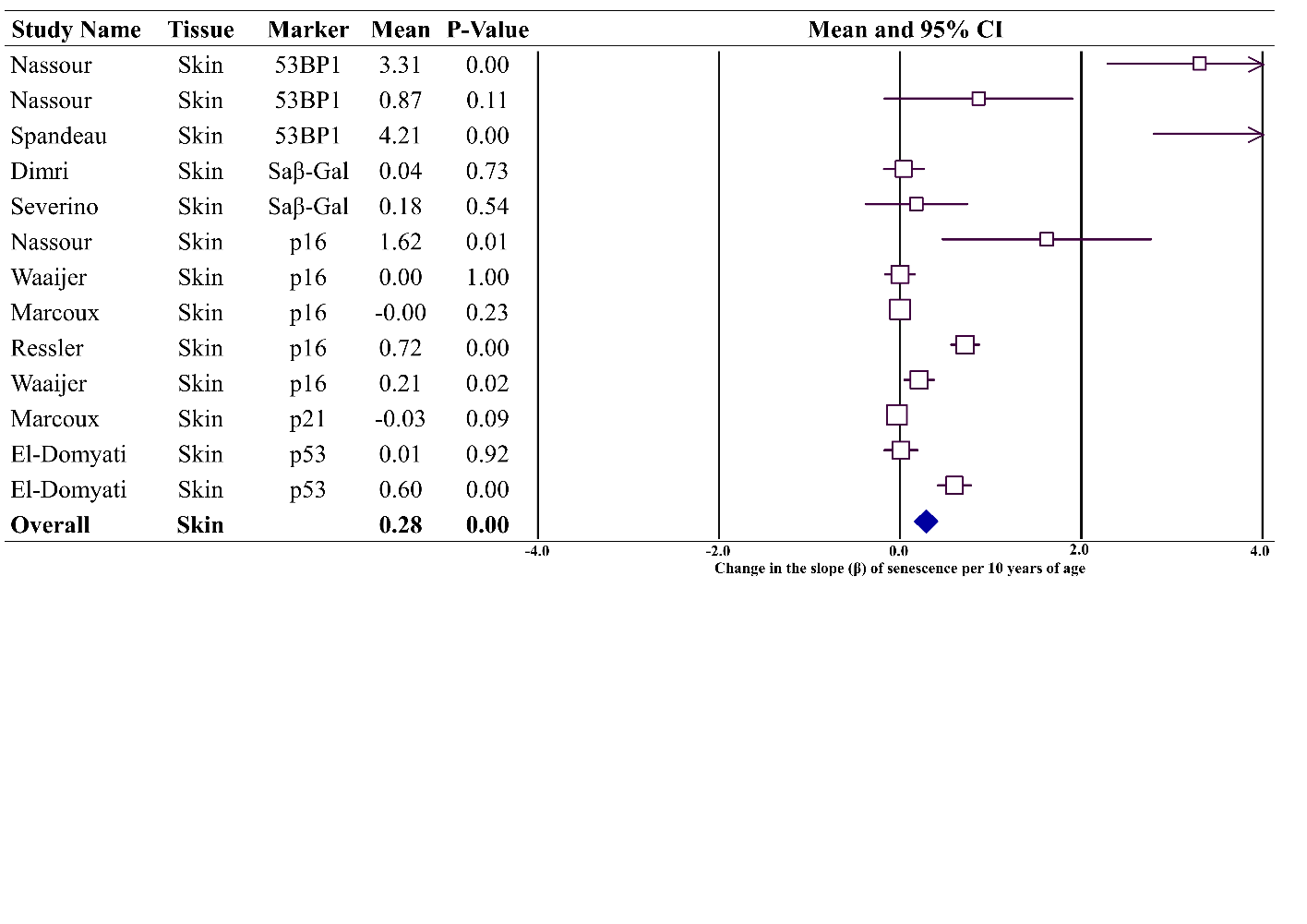


**Supplementary Figure 4A** Meta-Analysis for change in slope/10 years for senescence marker and age for Saβ-Gal


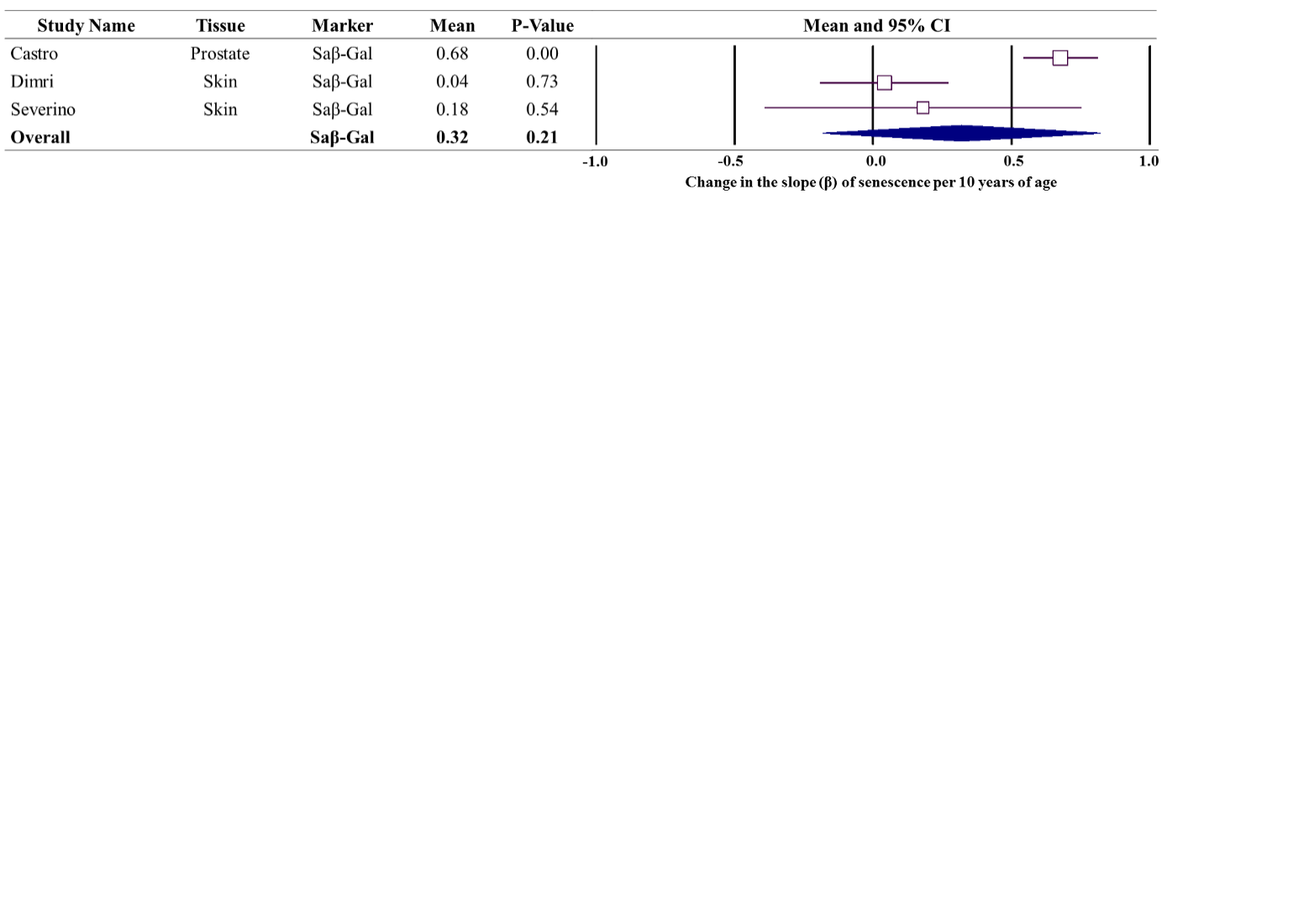


**Supplementary Figure 4B** Meta-Analysis for change in slope/10 years for senescence marker and age for the cell cycle regulators

**
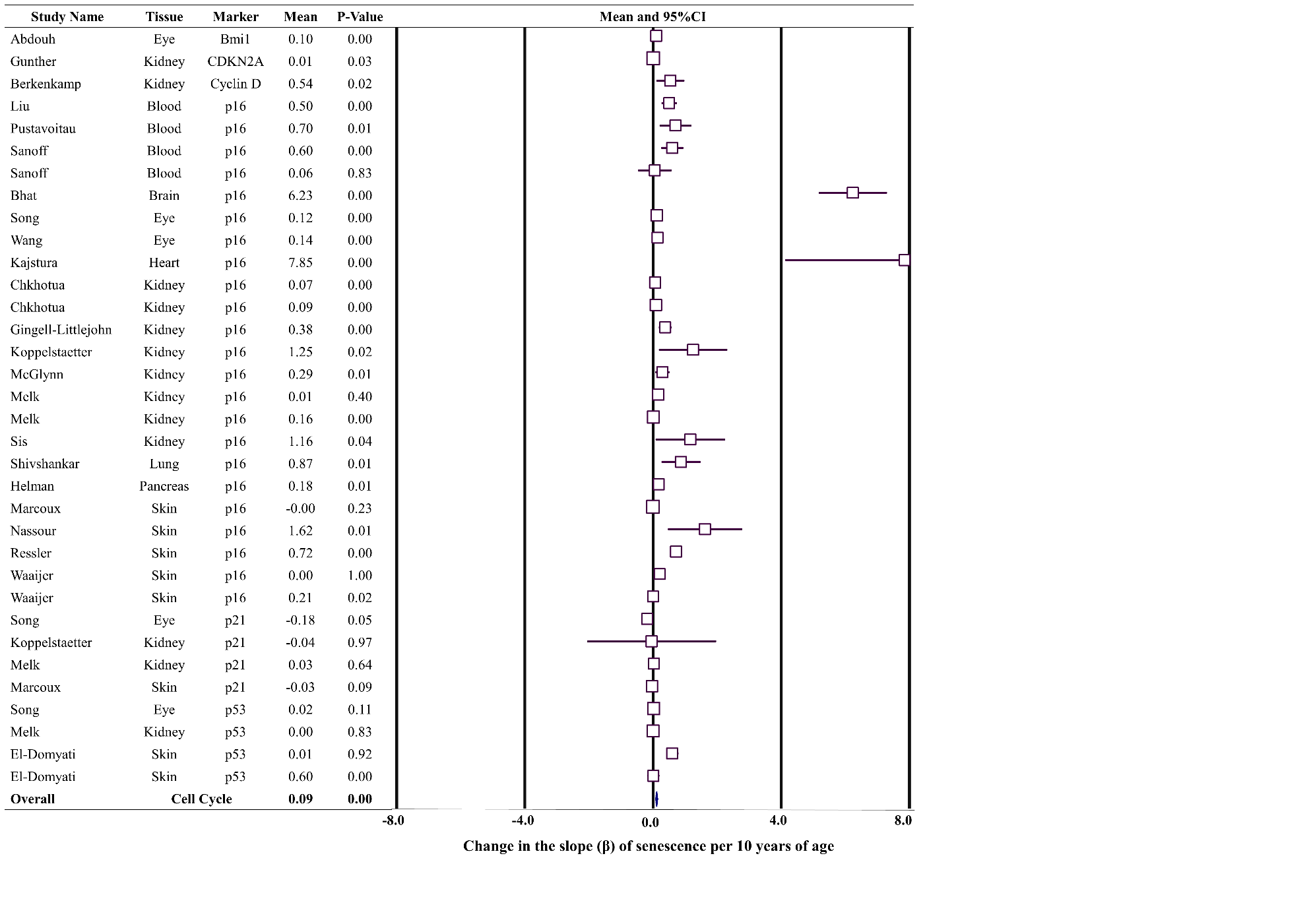
**

**Supplementary Figure 4C** Meta-Analysis for change in slope/10 years for senescence marker and age for the DNA damage markers.

**
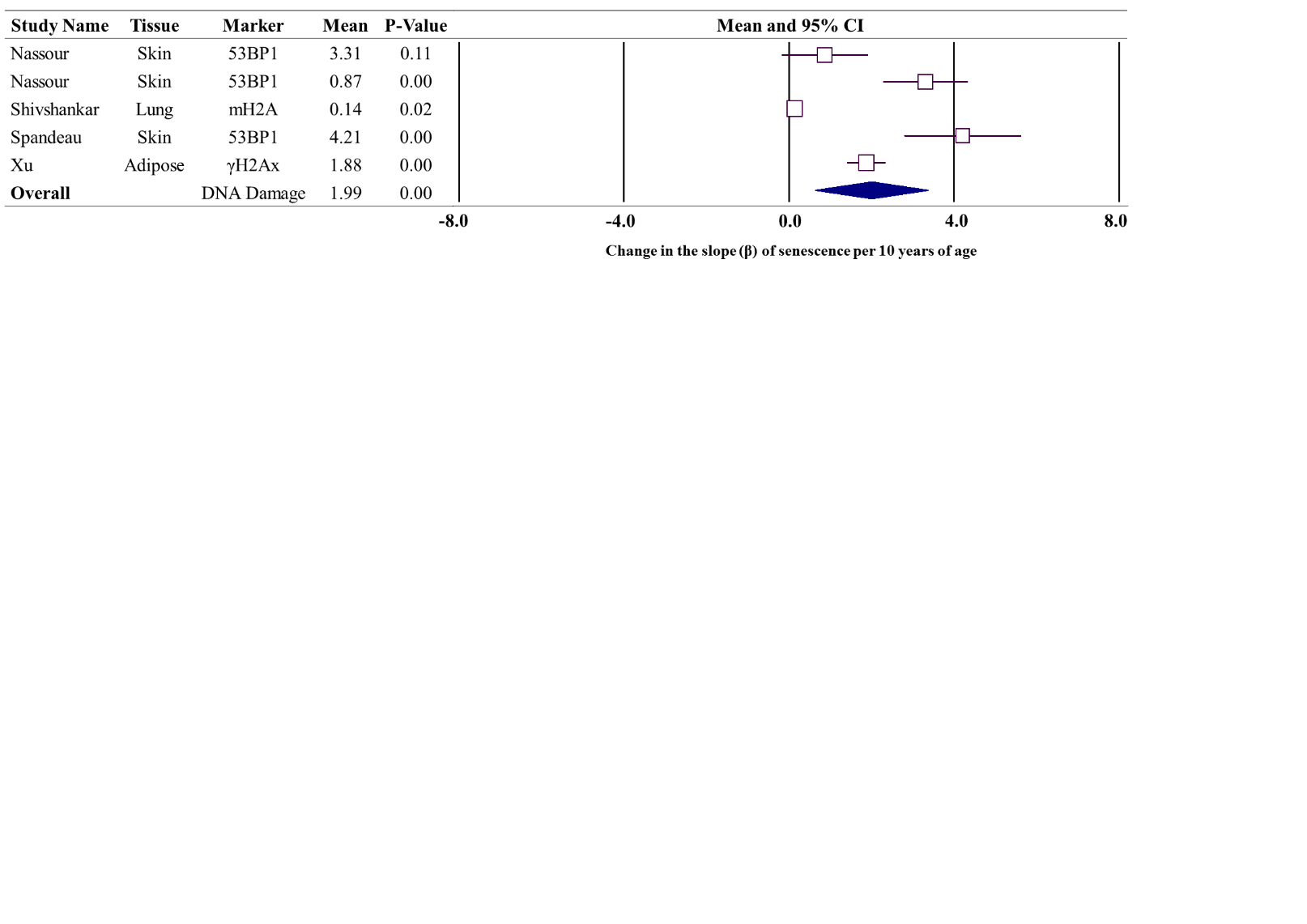
**

**Supplementary Figure 4D** Meta-Analysis for change in slope/10 years for senescence marker and age for the proliferation markers.

**
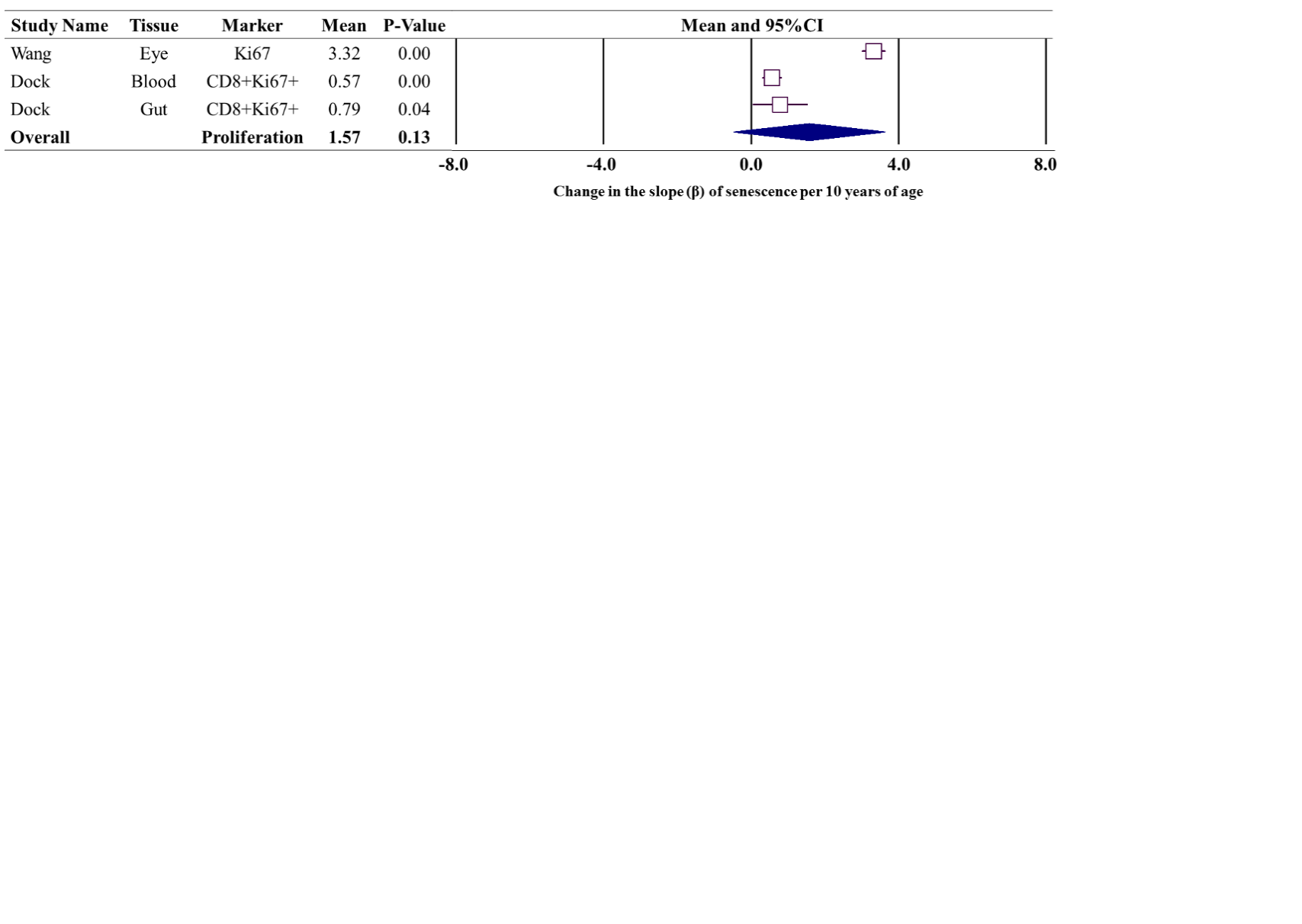
**

Reference

Abdouh, M., Chatoo, W., El, H. J., David, J., Ferreira, J., & Bernier, G. (2012). Bmi1 is down-regulated in the aging brain and displays antioxidant and protective activities in neurons. PLoS ONE, 7(2). <https://doi.org/10.1371/journal.pone.0031870>

Bartkova, J., Moudry, P., Hodny, Z., Lukas, J., Rajpert-De, M. E., & Bartek, J. (2011). Heterochromatin marks HP1, HP1alpha and H3K9me3, and DNA damage response activation in human testis development and germ cell tumours. International Journal of Andrology, 34(4 PART 2), e103–e113.

Berkenkamp, B., Susnik, N., Baisantry, A., Kuznetsova, I., Jacobi, C., Sörensen-Zender, I., … Schmitt, R. (2014). In vivo and in vitro analysis of age-associated changes and somatic cellular senescence in renal epithelial cells. PLoS ONE, 9(2), e88071. <https://doi.org/10.1371/journ>al.pone.0088071

Cao, S., Walker, G. B., Wang, X., Cui, J. Z., & Matsubara, J. A. (2013). Altered cytokine profiles of human retinal pigment epithelium: Oxidant injury and replicative senescence. Molecular Vision, 19, 718–728.

Castro, P., Giri, D., Lamb, D., & Ittmann, M. (2003). Cellular senescence in the pathogenesis of benign prostatic hyperplasia. Prostate, 55(1), 30–38. https://doi.org/10.1002/pros.10204

Choi, J., Shendrik, I., Peacocke, M., Peehl, D., Buttyan, R., Ikeguchi, E. F., … Benson, M. C. (2000). Expression of senescence-associated beta- galactosidase in enlarged prostates from men with benign prostatic hyperplasia. Urology, 56(1), 160–166. https://doi.org/10.1016/S0090-4295(00)00538-0

Dimri, G. P., Lee, X., Basile, G., Acosta, M., Scott, G., Roskelley, C., Medrano, E. E., Linskens, M., Rubelj, I., & Pereira-Smith, O., …1995). A biomarker that identifies senescent human cells in culture and in aging skin in vivo. Proceedings of the National Academy of Sciences of the United States of America, 92(20), 9363–9367. https://doi.org/10.1073/pnas.92.20.9363

Disayabutr, S., Kim, E. K., Cha, S.-I., Green, G., Naikawadi, R. P., Jones, K. D., … Wolters, P. J. (2016). miR-34 miRNAs regulate cellular senescence in type II alveolar epithelial cells of patients with idiopathic pulmonary fibrosis. PLoS ONE, 11(6), e0158367. https://doi.org/10.1371/journ al.pone.0158367

Dreesen, O., Chojnowski, A., Ong, P. F., Zhao, T. Y., Common, J. E., Lunny, D., … Colman, A. (2013). Lamin B1 fluctuations have differential effects on cellular proliferation and senescence. Journal of Cell Biology, 200(5), 605–617. https://doi.org/10.1083/jcb.20120 6121

El-Domyati, M. B., Attia, S., Saleh, F., Galaria, N., Ahmad, H., Gasparro, F. P., & Uitto, J. (2003). Expression of p53 in normal sun-exposed and protected skin (type IV–V) in different decades of age. Acta Dermato- Venereologica, 83(2), 98–104. https://doi.org/10.1080/00015 550310007427

Gingell-Littlejohn, M., McGuinness, D., McGlynn, L. M., Kingsmore, D., Stevenson, K. S., Koppelstaetter, C., … Shiels, P. G. (2013). Pretransplant CDKN2A expression in kidney biopsies predicts renal function and is a future component of donor scoring criteria. PLoS ONE, 8(7), e68133. https://doi.org/10.1371/journ al.pone.0068133

Going, J. J., Stuart, R. C., Downie, M., Fletcher-Monaghan, A. J., & Nicol, K. W. (2002). ‘Senescence-associated’ beta-galactosidase activity in the upper gastrointestinal tract. Journal of Pathology, 196(4), 394–400.

Günther, J., Resch, T., Hackl, H., Sattler, A., Ebner, S., Ritschl, P. V., … Kotsch, K. (2017). Identification of the activating cytotoxicity receptor NKG2D as a senescence marker in zero-hour kidney biopsies is indicative for clinical outcome. Kidney International, 91(6), 1447–1463. https://doi.org/10.1016/j.kint.2016.12.018

Helman, A., Klochendler, A., Azazmeh, N., Gabai, Y., Horwitz, E., Anzi, S., … Ben-Porath, I. (2016). p16(Ink4a)-induced senescence of pancreatic beta cells enhances insulin secretion. Nature Medicine, 22(4), 412–420. https://doi.org/10.1038/nm.4054

Justice, J. N., Gregory, H., Tchkonia, T., LeBrasseur, N. K., Kirkland, J. L., Kritchevsky, S. B., & Nicklas, B. J. (2017). Cellular senescence biomarker p16INK4a+ cell burden in thigh adipose is associated with poor physical function in older women. Journals of Gerontology. Series A, Biological Sciences and Medical Sciences, 73(7), 939–945. https://doi.org/10.1093/geron a/glx134

Kajstura, J., Gurusamy, N., Ogórek, B., Goichberg, P., Clavo-Rondon, C., Hosoda, T., … Anversa, P. (2010). Myocyte turnover in the aging human heart. Circulation Research, 107(11), 1374–1386. https://doi.org/10.1161/CIRCR ESAHA.110.231498

Kanavaros, P., Stefanaki, K., Rontogianni, D., Papalazarou, D., Sgantzos, M., Arvanitis, D., … Bai, M. (2001). Immunohistochemical expression of p53, p21/waf1, Rb, p16, cyclin D1, p27, Ki67, cyclin A, cyclin B1, bcl2, bax and bak proteins and apoptotic index in normal thymus. Histology and Histopathology, 16(4), 1005–1012.

Klement, K., Melle, C., Murzik, U., Diekmann, S., Norgauer, J., & Hemmerich, P. (2012). Accumulation of annexin A5 at the nuclear envelope is a biomarker of cellular aging. Mechanisms of Ageing and Development, 133(7), 508–522. https://doi.org/10.1016/j.mad.2012.06.003

Koppelstaetter, C., Schratzberger, G., Perco, P., Hofer, J., Mark, W., llinger, R., … Mayer, G. (2008). Markers of cellular senescence in zero hour biopsies predict outcome in renal transplantation. Aging Cell, 7(4), 491–497. https://doi.org/10.1111/j.1474-9726.2008.00398.x

Lang, A., Grether-Beck, S., Singh, M., Kuck, F., Jakob, S., Kefalas, A., … Piekorz, R. P. (2016). MicroRNA-15b regulates mitochondrial ROS production and the senescence-associated secretory phenotype through sirtuin 4/SIRT4. Aging (Albany NY), (3), 484–505. https://doi.org/10.18632/ aging.100905

Lee, J. H., Yoo, J. H., Oh, S. H., Lee, K.-Y., & Lee, K. H. (2010). Knockdown of moesin expression accelerates cellular senescence of human dermal microvascular endothelial cells. Yonsei Medical Journal, 51(3), 438–447. https://doi.org/10.3349/ymj.2010.51.3.438

Liu, J., Yang, J.-R., He, Y.-N., Cai, G.-Y., Zhang, J.-G., Lin, L.-R., … Xiao, H.-S. (2012). Accelerated senescence of renal tubular epithelial cells is associated with disease progression of patients with immunoglobulin A (IgA) nephropathy. Translational Research, 159(6), 454–463. https://doi.org/10.1016/j.trsl.2011.11.008

Liu, Y., Sanoff, H. K., Cho, H., Burd, C. E., Torrice, C., Ibrahim, J. G., … Sharpless, N. E. (2009). Expression of p16(INK4a) in peripheral blood T-cells is a biomarker of human aging. Aging Cell, 8(4), 439–448. https://doi.org/10.1111/j.1474-9726.2009.00489.x

Marchand, A., Atassi, F., Gaaya, A., Leprince, P., Le Feuvre, C., Soubrier, F., … Nadaud, S. (2011). The Wnt/beta-catenin pathway is activated during advanced arterial aging in humans. Aging Cell, 10(2), 220–232. https ://doi.org/10.1111/j.1474-9726.2010.00661.x

Marcoux, S., Le, O. N. L., Langlois-Pelletier, C., Laverdiere, C., Hatami, A., Robaey, P., & Beausejour, C. M. (2013). Expression of the senescence marker p16INK4a in skin biopsies of acute lymphoblastic leukemia survivors: A pilot study. Radiation Oncology, 8(1). https://doi.org/10.1186/1748-717X-8-252

McGlynn, L. M., Stevenson, K., Lamb, K., Zino, S., Brown, M., Prina, A., … Shiels, P. G. (2009). Cellular senescence in pretransplant renal biopsies predicts postoperative organ function. Aging Cell, 8(1), 45–51. https://doi.org/10.1111/j.1474-9726.2008.00447.x

Melk, A., Schmidt, B. M. W., Takeuchi, O., Sawitzki, B., Rayner, D. C., & Halloran, P. F. (2004). Expression of p16INK4a and other cell cycle regulator and senescence associated genes in aging human kidney. Kidney International, 65(2), 510–520. https://doi.org/10.1111/j.1523-1755.2004.00438.x

Morgan, R. G., Ives, S. J., Lesniewski, L. A., Cawthon, R. M., Andtbacka, R. H. I., Noyes, R. D., … Donato, A. J. (2013). Age-related telomere uncapping is associated with cellular senescence and inflammation independent of telomere shortening in human arteries. American Journal of Physiology – Heart and Circulatory Physiology, 305(2),

H251–H258. https://doi.org/10.1152/ajphe art.00197.2013

Nassour, J., Martien, S., Martin, N., Deruy, E., Tomellini, E., Malaquin, N., … Abbadie, C. (2016). Defective DNA single-strand break repair is responsible for senescence and neoplastic escape of epithelial cells. Nature Communications, 7, 10399. https://doi.org/10.1038/ncomms10399

Nielsen, G. P., Stemmer-Rachamimov, A. O., Shaw, J., Roy, J. E., Koh, J., & Louis, D. N. (1999). Immunohistochemical survey of p16INK4A expression in normal human adult and infant tissues. Laboratory Investigation, 79(9), 1137–1143.

Nuciforo, P. G., Luise, C., Capra, M., Pelosi, G., & Di Fagagna, F. D. (2007). Complex engagement of DNA damage response pathways in human cancer and in lung tumor progression. Carcinogenesis, 28(10), 2082–2088. https://doi.org/10.1093/carci n/bgm108

Pustavoitau, A., Barodka, V., Sharpless, N. E., Torrice, C., Nyhan, D., Berkowitz, D. E., … Walston, J. D. (2016). Role of senescence marker p16 INK4a measured in peripheral blood T-lymphocytes in predicting length of hospital stay after coronary artery bypass surgery in older adults. Experimental Gerontology, 74, 29–36. https://doi.org/10.1016/j.exger.2015.12.003

Ressler, S., Bartkova, J., Niederegger, H., Bartek, J., Scharffetter-Kochanek, K., Jansen-Durr, P., & Wlaschek, M. (2006). p16INK4A is a robust in vivo biomarker of cellular aging in human skin. Aging Cell, 5(5), 379–389

Rossman, M. J., Kaplon, R. E., Hill, S. D., McNamara, M. N., Santos-Parker, J. R., Pierce, G. L., … Donato, A. J. (2017). Endothelial cell senescence with aging in healthy humans: Prevention by habitual exercise and relation to vascular endothelial function. American Journal of Physiology. Heart and Circulatory Physiology, 313(5), H890–H895. https://doi.org/10.1152/ajphe art.00416.2017

Sanoff, H. K., Deal, A. M., Krishnamurthy, J., Torrice, C., Dillon, P., Sorrentino, J., … Muss, H. B. (2014). Effect of cytotoxic chemotherapy on markers of molecular age in patients with breast cancer. Journal of the National Cancer Institute, 106(4), dju057. https://doi.org/10.1093/jnci/dju057

Schonland, S. O., Lopez, C., Widmann, T., Zimmer, J., Bryl, E., Goronzy, J. J., & Weyand, C. M. (2003). Premature telomeric loss in rheumatoid arthritis is genetically determined and involves both myeloid and lymphoid cell lineages. Proceedings of the National Academy of Sciences of the United States of America, 100(23), 13471–13476. https://doi.org/10.1073/pnas.22335 61100

Severino, J., Allen, R. G., Balin, S., Balin, A., & Cristofalo, V. J. (2000). Is beta-galactosidase staining a marker of senescence in vitro and in vivo? Experimental Cell Research, 257(1), 162–171.

Shivshankar, P., Boyd, A. R., Le Saux, C. J., Yeh, I.-T., & Orihuela, C. J. (2011). Cellular senescence increases expression of bacterial ligands in the lungs and is positively correlated with increased susceptibility to pneumococcal pneumonia. Aging Cell, 10(5), 798–806. https://doi.org/10.1111/j.1474-9726.2011.00720.x

Spandau, D. F., Lewis, D. A., Somani, A. K., & Travers, J. B. (2012). Fractionated laser resurfacing corrects the inappropriate UVB response in geriatric skin. Journal of Investigative Dermatology, 132(6), 1591–1596. https://doi.org/10.1038/jid.2012.29

Werner, C., Fürster, T., Widmann, T., Pöss, J., Roggia, C., Hanhoun, M., … Laufs, U. (2009). Physical exercise prevents cellular senescence in circulating leukocytes and in the vessel wall. Circulation, 120(24), 2438–2447. https://doi.org/10.1161/CIRCU LATIO NAHA.109.861005

Xu, M., Tchkonia, T., Ding, H., Ogrodnik, M., Lubbers, E. R., Pirtskhalava, T., Kirkland, J. L. (2015). JAK inhibition alleviates the cellular senescence-associated secretory phenotype and frailty in old age. Proceedings of the National Academy of Sciences of the United States of America, 112(46), E6301–E6310. https://doi.org/10.1073/pnas.15153 86112

Zang, X., Wang, Y., & Chen, P. (2012). High expression of p16INK4a and low expression of Bmi1 are associated with endothelial cellular senescence in the human cornea. Molecular Vision, 18, 803–815.

**Supplementary Figure 4E** Meta-Analysis for change in slope/10 years for senescence marker and age for the senescence associated secretory phenotype (SASP) markers.


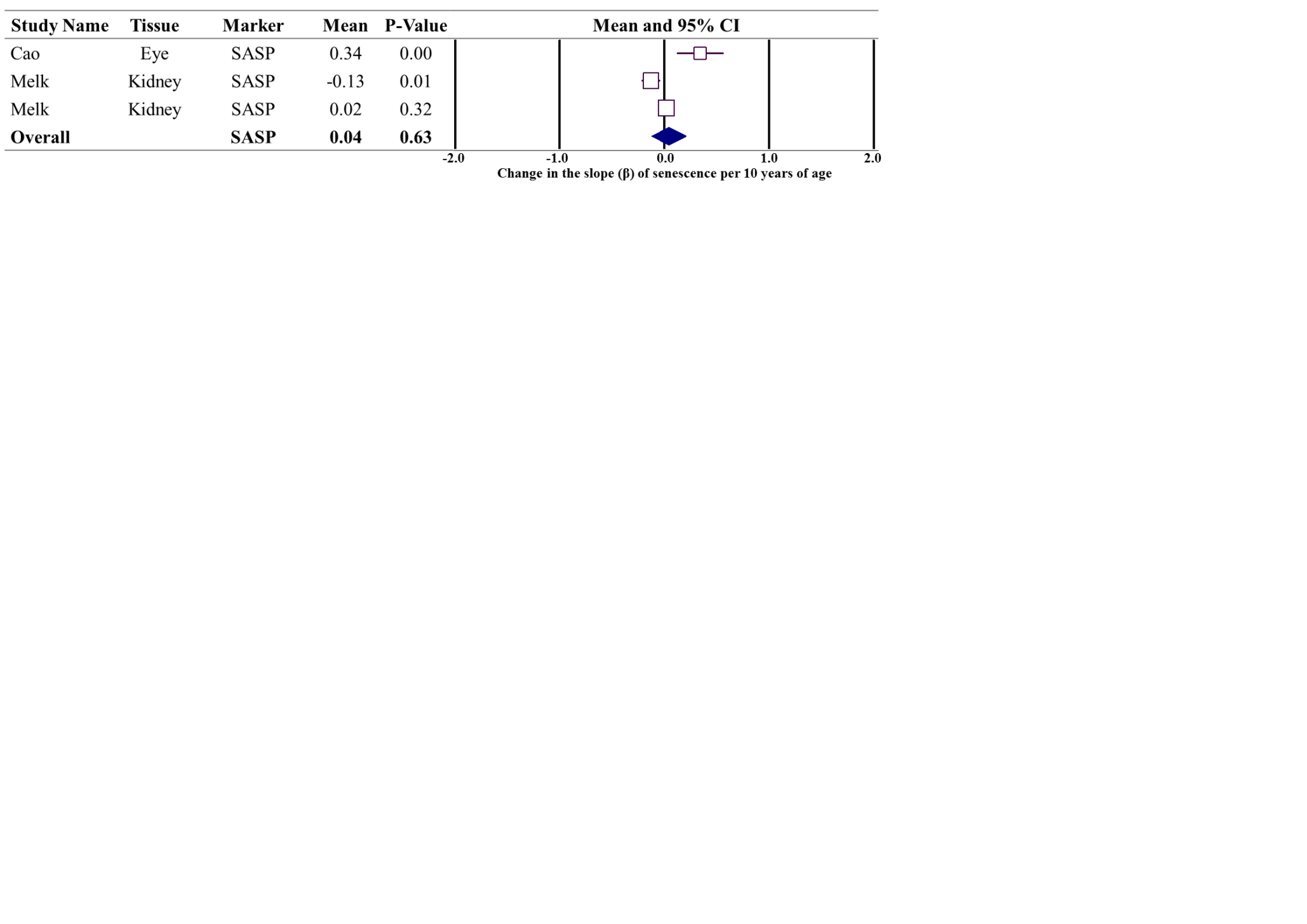

Supplement: Supplementary file 1 [file ACEL-19-e13083-s001.docx]
